# Movement characteristics impact decision-making and vice versa

**DOI:** 10.1038/s41598-023-30325-4

**Published:** 2023-02-25

**Authors:** Thomas Carsten, Fanny Fievez, Julie Duque

**Affiliations:** CoActions Lab, Institute of Neuroscience, Université Catholique Louvain, Tour Bernard Tower, 2d Floor, Ave Hippocrate 54, 1200 Bruxelles, Belgium

**Keywords:** Human behaviour, Decision

## Abstract

Previous studies suggest that humans are capable of coregulating the speed of decisions and movements if promoted by task incentives. It is unclear however whether such behavior is inherent to the process of translating decisional information into movements, beyond posing a valid strategy in some task contexts. Therefore, in a behavioral online study we imposed time constraints to either decision- or movement phases of a sensorimotor task, ensuring that coregulating decisions and movements was not promoted by task incentives. We found that participants indeed moved faster when fast decisions were promoted and decided faster when subsequent finger tapping movements had to be executed swiftly. These results were further supported by drift diffusion modelling and inspection of psychophysical kernels: Sensorimotor delays related to initiating the finger tapping sequence were shorter in fast-decision as compared to slow-decision blocks. Likewise, the decisional speed-accuracy tradeoff shifted in favor of faster decisions in fast-tapping as compared to slow-tapping blocks. These findings suggest that decisions not only impact movement characteristics, but that properties of movement impact the time taken to decide. We interpret these behavioral results in the context of embodied decision-making, whereby shared neural mechanisms may modulate decisions and movements in a joint fashion.

## Introduction

When animals hunt, they may have to decide quickly whether turning left or right allows cutting into their prey’s path, before putting this plan into action. Traditional views have understood this behavior as relying on at least two functionally distinct brain processes^1,2^: ‘Deciding’ regards the comparison and selection between expected outcomes based on sensory information, whereas ‘moving’ reflects the interaction with the environment to achieve the favored outcome. These two processes have been associated with different brain structures^3,4^ and different neurophysiological signatures^5–7^. Perhaps as a result of this predominant view, the interplay between decision-making and movement has not been studied extensively^8^.

Over the course of the past ten years or so, increased interest in the interaction between decisions and movements has generated findings which challenge the view that these processes operate independently. For instance, properties of decisions such as reward expectancy^9,10^, choice preference^11–13^, conflict anticipation^14,15^ and time pressure^16–19^ systematically change activity in motor areas of the brain, and alter kinematics of movements expressing those decisions^10,20–22^. Likewise, kinematic properties of movements such as required time^23,24^ and energetic costs^25–27^ are factored into decisions. Taken together, these findings challenge the view that decisions and movements pose fully functionally distinct aspects of behavior^28^.

Such interplay may reflect adjustments concerning both decisions and movements in order to increase the net payoff of behavior. For example, biomechanical costs of movement may be weighed against its expected outcome in order to justify its effort expenditure^27,29,30^. Similarly, under time pressure, reducing both the time to decide and to move shortens the overall time required to respond^21^. Taken together, animals, including humans, likely aim to maximize ‘capture rate’, which is the amount of obtained rewards minus the effort exerted to acquire these, divided by the total time spent^10,22,24,31^. Hence, minimizing the time and effort required to obtain reward likely leads to adaptations concerning both decisions and movements.

If animal behavior in terms of decisions and movements serves a common function to secure capture rate and hence survival, then a close consensus between decisions and movements may be naturally promoted by the organization of the sensorimotor system^32^. Rather than assuming independent brain systems to decide and to move, it seems more accurate to assume a gradual shift in functionality between anatomically distinct brain areas, transforming abstract-decisional considerations of future consequences to their concrete implementation through movement^8,33–35^. Given a reciprocal, continuous and interactive communication between brain areas alongside this gradient^35,36^, a distributed consensus about the preferred course of action may be reached, reflecting both abstract decisional considerations of future consequences and concrete motor requirements for the implementation of the response^34^. In addition, common drivers such as the basal ganglia^37–39^ and the locus coeruleus-noradrenergic system^18,19,40^ may take broad influence on behavior as a whole, modulating decisional and movement-related aspects of behavior in a joint fashion. For these reasons, the semantic distinction between decisions and movements may be somewhat artificial and not be fully represented in brain function^32^. Instead, movements may be considered *expressed* or *embodied* decisions in that they establish preferred outcomes through interaction with the environment. From this *embodied* *decision-making* perspective, a close functional relationship between decisions and movements is hence expected, whereby movement kinematics depend on decisional information and the integration of information into decisions is fundamentally constrained by movement requirements.

It was the goal of this study to seek behavioral evidence for embodied decision-making, which has remained largely theoretical despite of its appeal. Although previous studies in this regard largely demonstrated how decisions and (preparation of) movements change under time pressure^17–19,21,22,41–43^, the nature of these tasks actively promoted a joint reduction of time taken to decide and move, to reduce overall response times. However, from an embodied decision-making perspective a mutual dependence of decisions and movements may reflect an inherent property of the sensorimotor system. It should therefore be observable even if there is no task incentive linking decisions and movements. Likewise, it implies that such interdependence sustains even when time pressure is limited towards *either* decisions or movements, rather than towards both at the same time as in previous studies.

With a behavioral online study run on 62 participants, we tested two predictions grounded in the *embodied* *decision-making* framework. As a first hypothesis (H1), people should tend to move quicker if they have less time to decide, even if such behavior is not required, nor advantageous. As a second hypothesis (H2), conversely, people should tend to decide faster as they need to perform faster movements, again even if not profitable. We found such evidence by imposing time constraints either to decision phases or movement phases of a sensorimotor task. Participants indeed reduced both the time to decide and to move although time constraints regarded only either decisions or movements. These findings were consistent across three complementary approaches of analysis: (1) We compared behavioral endpoint measures statistically, (2) fit behavior to drift–diffusion models, and (3) compared shapes of psychophysical kernels, reflecting the relationship between momentary decisional evidence and the participant’s decisions. Each approach separated shifts in the time taken to decide from sensorimotor delays related to initiating the movement in a different way, speaking for the robustness of these findings. Remarkably, most participants who displayed larger instructed time savings in either decision or movement duration also speeded up corresponding movements or decisions more, respectively, although not profitable in this task. These findings support the idea that decisions and movements are not fully functionally distinct brain processes, but condition each other even when not required in the context of the task.

## Results

**Figure 1:**
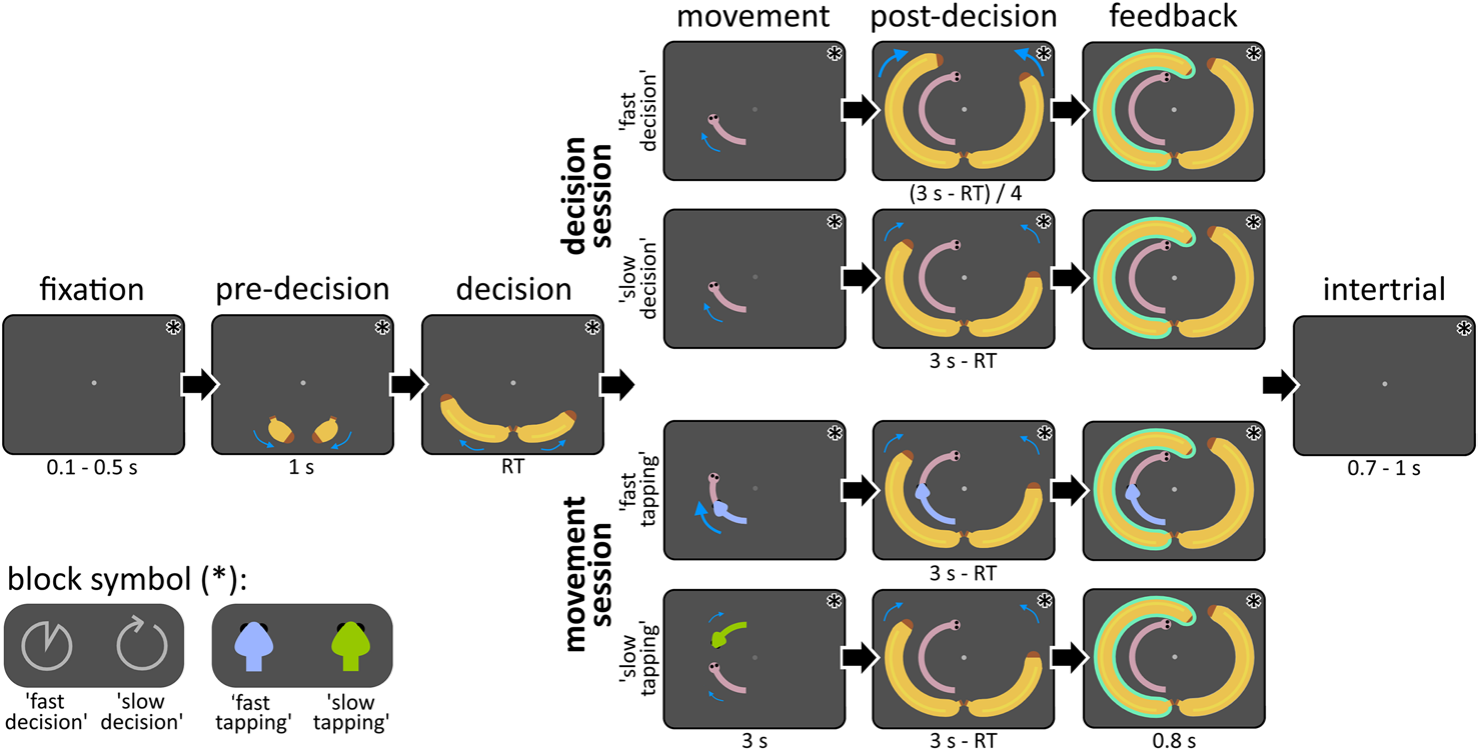
Trial structure. After some time of fixation, baby bananas moved symmetrically into position below the fixation circle, telegraphing the onset of the decision phase. The latter was characterized by asymmetrical growth of bananas, during which participants had to indicate which banana would outgrow the other with a key press. After this decision, the same key had to be pressed four more times in the movement phase to move a caterpillar into the corresponding direction. Here, the required tapping speed depended on the experimental condition: Fast-tapping blocks required participants to tap fast to avoid a chasing snake, whereas slow-tapping blocks required slow tapping to avoid a retreating snake. In contrast, fast-decision and slow-decision conditions had no specific finger tapping speed requirements but required to put the emphasis on either decision speed or accuracy, respectively. In the post-decision phase, bananas finished their growth trajectories until a full circle was covered. Here, bananas grew at same speed as in the decision phase, with the exception of the fast-decision condition, during which bananas grew four times faster. Afterwards, decision feedback was given by highlighting the chosen banana either in green (correct) or red (incorrect). A final intertrial interval preceded the next trial. Blue arrows depict movement trajectories of the stimuli, with thicker and longer arrows reflecting faster movement. Arrows are shown for illustrative purposes and were not visible to participants. Symbols were shown in the upper right corner of the screen (indicated by asterisks), as constant reminder of one of the four experimental block conditions. *s* seconds, *RT* reaction time.

Sixty-two participants performed two separate sessions of a behavioral online experiment from home. We administered an adaptation of the Tokens task, which has been widely used to investigate the effect of time pressure on sensorimotor processes in humans^22,24,44–46^. Each trial consisted of a dedicated decision and movement phase, which allowed to measure durations of decisions and movements separately (see Fig. 1). In the decision phase, participants decided which one of two bananas would outgrow the other; both grew at a non-constant speed over time. Their decision had to be indicated by a left- or rightward key press, which ended the decision phase and initiated the subsequent movement phase. Here, participants continued tapping this key to move a caterpillar upwards. The caterpillar moved faster with faster key tapping, however participants were made aware that the duration of the movement phase was fixed throughout the entire experiment. This basic task template was modified in a ‘decision session’ to encourage fast or slow decisions in separate blocks, with no constraints on tapping speed: In short, for each block a monetary bonus was provided for completing more trials by deciding faster or completing more trials correctly by deciding more carefully. In a separate ‘movement session’, fast or slow finger tapping was required, with no restrictions on decision speed. Here, tapping speed was constrained to a minimum (fast-tapping) or to a maximum (slow-tapping) through continuous visual feedback in the movement phase. To test H1 and H2, in all subsequent analyses decision durations were separated from sensorimotor delays related to initiating the finger tapping sequence. Sensorimotor delays were estimated for behavioral data analysis in a separate Simple Reaction Time (SRT) task, which had no decisional component, but kept all other task aspects similar, including movement constraints. Decision durations were computed per experimental condition by subtracting SRTs from reaction times in the main task^22,24,39,45–48^. Comparing finger tapping speed in the movement phase between blocks of fast and slow decisions and comparing decision duration in the decision phase between blocks of fast and slow tapping allowed to test the hypothesis that the duration of decisions and movements impact each other, even if not profitable in the context of the task. Results are reported by rounding *p*-values lower than 0.01 to the next higher decimal.

### When participants are required to decide faster, they also move faster

**Figure 2:**
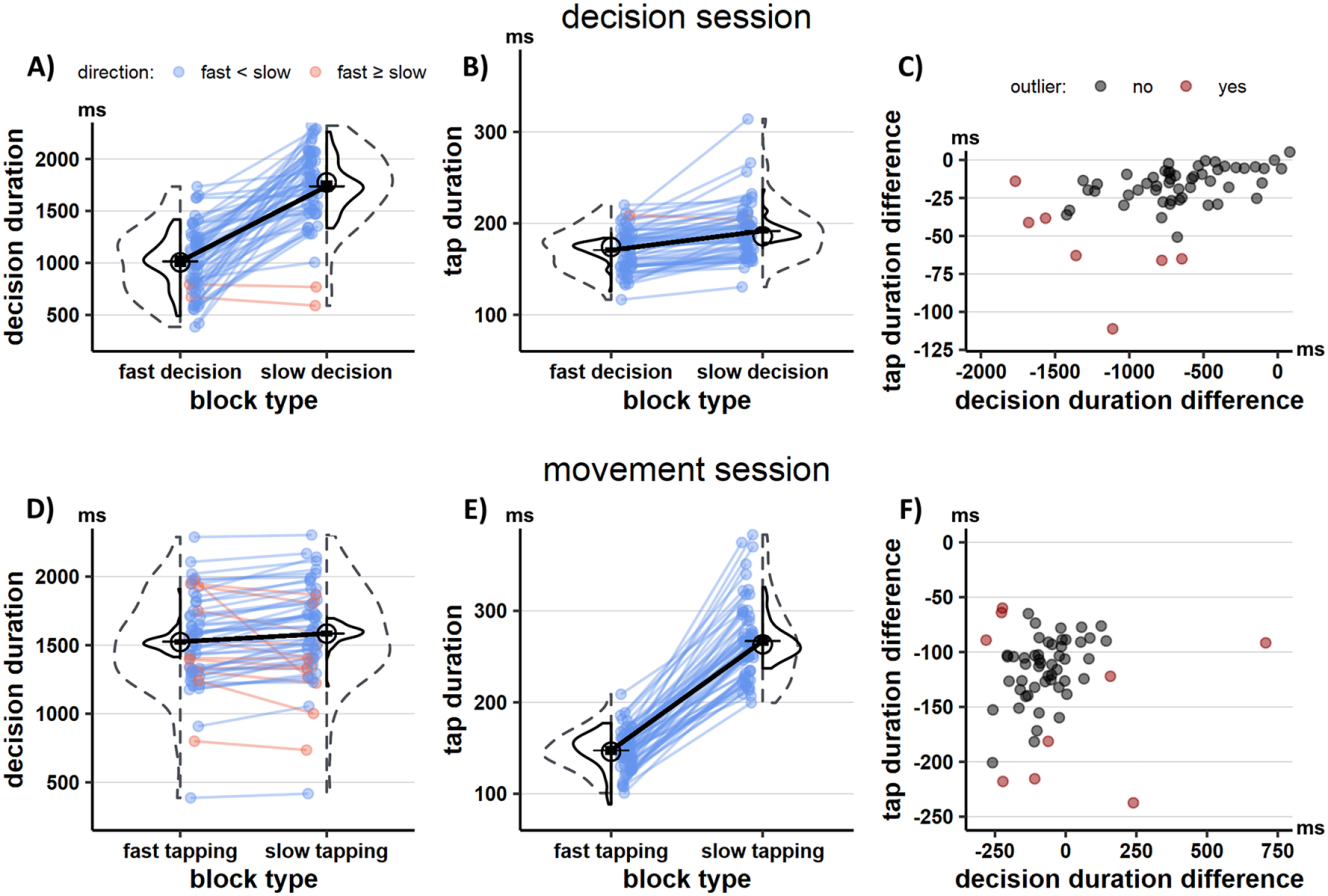
Behavioral results. The upper graphs (**A**–**C**) depict data for the decision session; three lower graphs (**D**–**F**) are for the movement session. (**A**) As instructed, participants changed their decision duration between blocks in the decision session. (**B**) Although not profitable in the context of the task, participants tapped faster in fast-decision blocks as compared to slow-decision blocks. (**C**) There was a monotonic relationship between the instructed change in decision duration and the resulting change in tap duration across participants. This monotonic relationship was significant both when considering the entire sample, as well as the sample after removing outliers (red data points). (**D**) Although not profitable in the context of the task, participants decided faster in fast-tapping blocks as compared to slow-tapping blocks. (**E**) As instructed, participants changed their tap duration between blocks in the movement session. (**F**) There was no monotonic relationship between the instructed change in tap duration and the resulting change in decision duration across participants when considering the whole sample. However, there was a significant relationship after removing outliers (red data points). (**A**,**B**,**D**,**E**) Individual data is shown as colored points connected by a line, with blue data indicating individuals with numerically lower values in fast as compared to slow decision or movement blocks and orange indicating the opposite. Dashed histograms indicate the distribution of data points for each condition. Solid histograms reflect the same distributions after centering subjects by replacing individual subject means with the group mean to remove between-subjects variance unrelated to the experimental manipulation^49^. The average effect on the group level is shown by plotting the mean (black horizontal lines), connected by a black line. Black bars around the mean reflect its within-subject 95% confidence interval^49^. The group median of each condition is shown as circle.

Participants followed the instructions consistently and adjusted their decision duration in fast and slow decision-blocks. As seen in Fig. 2A, participants took about 719 ms (95%-Confidence Interval [CI] 592–772 ms) less to decide which banana to choose in fast-decision (median = 1006 ms, Median Absolute Deviation [MAD] = 355 ms) as compared to slow-decision blocks (median = 1774 ms, *MAD* = 347 ms, *S* = 2, *p* = 10^–13^). This came at the price of reducing probability of making the correct decision by 13% (CI 11–14%) from a median of 86% (*MAD* = 5%) in slow-decision to 71% (*MAD* = 7%) in fast-decision blocks (*S* = 1, *p* = 10^–15^), reflecting a shift in speed-accuracy tradeoff (see Supplemental Fig. S1A).

As predicted with H1, this reduction in decision duration came with increased tapping speed in the movement phase following the decision (see Fig. 2B). The average duration of the four individual key taps was reduced by circa 16 ms (CI 12 to 20 ms) from slow-decision (*median* = 186 ms, *MAD* = 27 ms) to fast-decision blocks (*median* = 174 ms, *MAD* = 26 ms, *S* = 1, *p* = 10^–15^). This experimental effect was significant for each of the four finger taps in the movement phase (all *p*’s ≤ 10^–9^, see Supplemental Fig. S2A). Remarkably, Fig. 2C shows that those participants with the largest reduction in decision duration were also those who increased tapping speed the most between slow and fast decision-blocks (*t* = 4.81, *p* = 10^–4^, *r*_*s*_ = 0.54 and CI 0.31–0.71). A similar monotonic relationship was observed after removing seven influential values from this analysis (red dots in Fig. 2C; *t* = 4.00, *p* = 10^–3^, *r*_*s*_ = 0.50 and CI 0.24–0.69). Virtually all finger taps in the movement phase of fast-decision (*median* = 100%, *MAD* = 0%) and slow-decision (*median* = 100%, *MAD* = 0%) blocks were performed correctly (i.e., the four finger taps were executed within the three-second timeout), with no difference between blocks (*S* = 6, *p* > 0.99, see Supplemental Fig. S1B).

These behavioral results were supported by drift diffusion models. These models assume that reaction times in the decision phase of the task reflect the duration of the sensorimotor process covering both the decision and sensorimotor delays related to executing the first finger tap used for reporting the decision. By fitting different candidate models to behavior, it can hence be determined whether the experimental manipulation of decision speed also likely affected the speed with which the motor response in the decision phase was prepared and executed, above and beyond the finger taps following in the movement phase, which were analyzed by regular means (see previous paragraph). We hypothesized that behavior was best accounted for by a model, which allowed the *decision* *threshold* (as proxy for shifts in the decisional speed-accuracy tradeoff) and the *non-decision* *time* (as proxy for sensorimotor delays related to initiating the first finger tap reporting the decision) to differ between experimental conditions. This model was compared to simpler models fixing either the *decision* *threshold*, the *non-decision* *time* or both across experimental conditions (see Supplemental Table S1). It was also compared to a more complex model, which, in addition to the *decision* *threshold* and *non-decision* *time*, allowed the *drift* *rate* (as proxy for the efficiency with which a correct decision can be made) to differ between experimental conditions. Models were compared based on the bias-corrected Bayesian Predictive Information Criterion (BPIC)^50,51^. The most complex model, which allowed the *decision* *threshold*, *non-decision* *time* and *drift* *rate* to differ between experimental conditions, described behavior best (see Table S1). The *decision* *threshold* was significantly lower in fast-decision blocks than in slow-decision blocks (Probability > 99.975%, i.e., exceeding 3999 out of 4000 posterior samples). In line with H1, the *non-decision* *time* was also significantly reduced (Probability > 99.975%, see Table 1). Although not expected, the *drift* *rate* was also lowered (Probability > 99.975%), indicating that choosing under time pressure let to worse decision performance beyond deliberate shifts in speed-accuracy tradeoff ^52^.

**Table 1:** Estimated drift diffusion model parameters per experimental condition.

	Decision threshold		Non-decision time		Drift rate	
	Mean	CI	Mean	CI	Mean	CI
Slow decision (intercept)	3.17	3.02	0.73	0.65	0.97	0.90
		3.34		0.82		1.04
Fast decision (difference)	− 1.04	− 1.22	− 0.35	− 0.44	− 0.42	− 0.49
		− 0.85		− 0.25		− 0.34
Slow tapping (intercept)	2.87	2.73	0.80	0.72	0.91	0.85
		3.01		0.89		
Fast tapping (difference)	− 0.11	− 0.21	− 0.05	− 0.11		0.98
		− 0.01		0.01		

Parameter values are based on group posteriors of the best-fitting drift diffusion models of each session. Credible intervals (CI) cover 2.5% and 97.5% percentiles of the respective posterior distribution. To estimate within-subject effects, an intercept was fitted to the slow condition and the difference thereof was fitted to the fast condition of the respective session. For the movement session, only one *drift* *rate* was fitted for both experimental conditions, as this provided better model fit than the full model (see Table S1).

### When participants are required to move faster, they also decide faster

As shown in Fig. 2E, participants also followed the instructions consistently in the movement session. Average duration of each of the four key taps in the movement phase was circa 111 ms faster (CI 104–125 ms) when fast tapping (*median* = 146 ms, *MAD* = 22 ms) was required as compared to slow tapping (*median* = 263 ms, *MAD* = 45 ms, *S* = 0, *p* = 10^–17^). Each single finger tap was consistently faster when fast tapping was required as compared to slow tapping (all *p* ≤ 10^–16^, see Supplemental Fig. S2B). Hence, the experimental manipulation was effective in shifting tapping speed of participants between blocks. The proportion of trials in which participants performed tapping movements correctly was high, but it was significantly lower in fast-tapping (*median* = 93%, *MAD* = 4%) as compared to slow-tapping blocks (*median* = 100%, *MAD* = 0%, *S* = 1, *p* = 10^–15^, see Fig. S1D), consistent with the higher motor control requirement associated with the former condition.

We then tested the hypothesis H2 that decision duration changes with tapping speed. As predicted and shown in Fig. 2D, decisions were circa 89 ms (CI 48–108 ms) faster in fast-tapping blocks (*median* = 1522 ms, *MAD* = 317 ms) as compared to slow-tapping blocks (*median* = 1584 ms, *MAD* = 329 ms, *S* = 12, *p* = 10^–5^). The probability of making a correct decision was however not significantly different between slow-tapping blocks (*median* = 80%, *MAD* = 6%) and fast-tapping blocks (*median* = 80%, *MAD* = 6%, *S* = 24, *p* = 0.19, see Supplemental Fig. S1C). Across all participants, we did not find that those reducing decision duration the most were also those who speeded up tapping the most between blocks (see Fig. 2F; *t* = 1.46, *p* = 0.15, *r*_*s*_ = 0.19, *CI* = − 0.07 to 0.43). However, such monotonic relationship was present after removing nine influential values (red dots in Fig. 2F; *t* = 3.03, *p* = 0.01, *r*_*s*_ = 0.40, CI 0.13–0.62). This suggests that faster tapping is preceded by proportionally faster decisions across most, but not all participants.

Drift diffusion modelling corroborated the interpretation that requirements to tap fast shortened decisions. Using the same model comparison approach as for the decision session, out of the five candidate models, the hypothesized model fit behavioral data best. This hypothesized model allowed the *decision* *threshold* and the *non-decision* *time* to differ between experimental conditions (see Table S1), taken as proxies for shifts in decisional speed-accuracy tradeoff and sensorimotor delays related to initiating the finger tapping sequence, respectively. In line with H2, the *decision* *threshold* was significantly reduced in fast-tapping blocks (Probability = 98.30%), supporting the interpretation that movement requirements reduced the time taken to decide. The reduction in *non-decision* *time* did not reach conventional levels of significance (Probability = 95.60%), potentially since experimental constraints on tapping speed regarded the movement phase (visualized as chasing or retreating snakes, see Fig. 1), but not the first tap during the decision phase, which was modelled.

In the Supplemental Material, we show that the SRT task was well-suited to estimate sensorimotor delays since tapping behavior was highly comparable between SRT and main experimental tasks. We also demonstrate that the overall high task performance improved throughout each session, speaking against a decline in task engagement. Moreover, best-performing participants showed clear coregulation effects, speaking against the idea that these findings were driven by a lack of motivation. Likewise, coregulation of decisions and movements was not affected by individual understanding of instructions, suggesting that these were not a determining factor. Trial difficulty was also not critical for observing coregulation effects; these were mostly consistent across decisions of various difficulty.

### Psychophysical kernels are consistent with behavioral and modelling results

**Figure 3:**
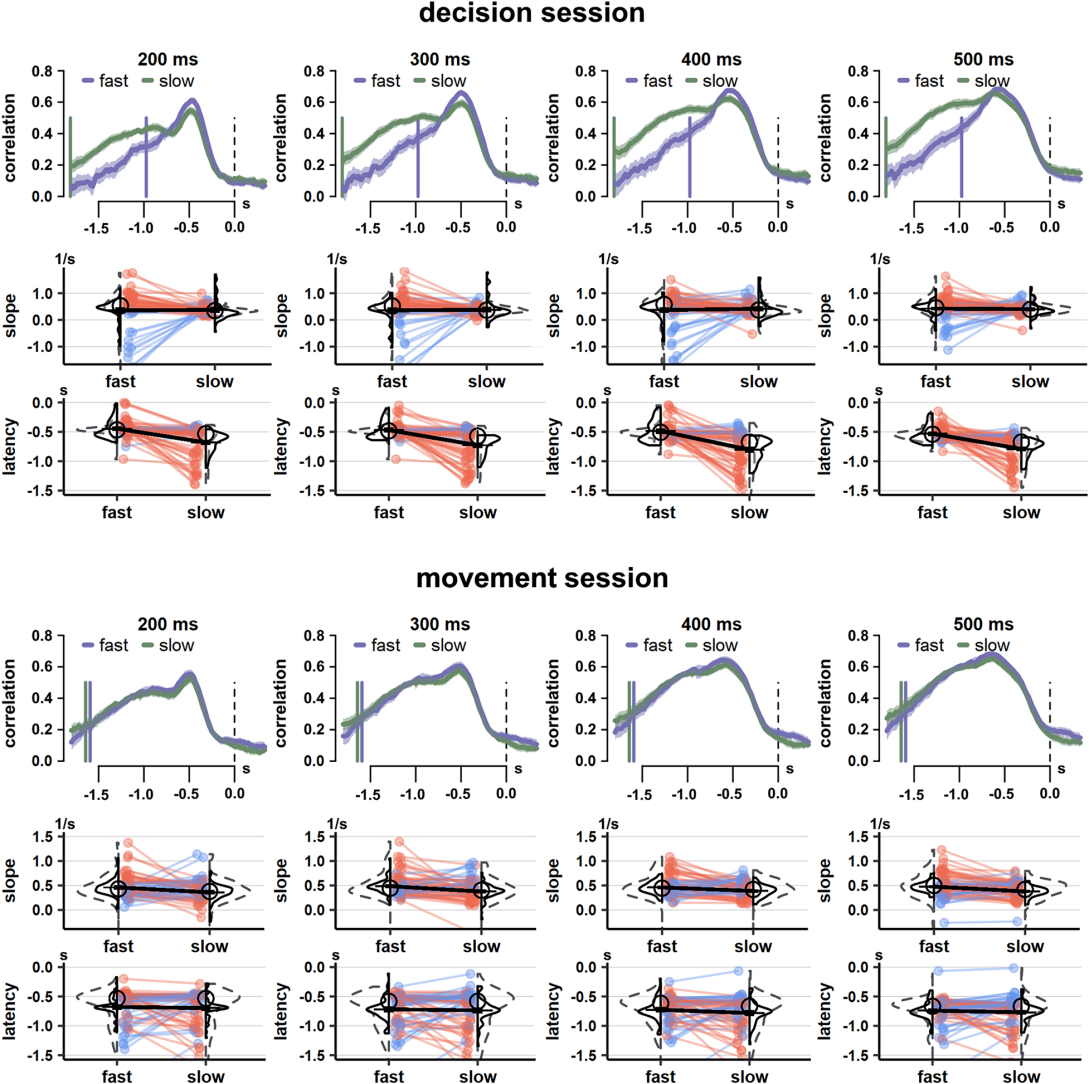
Psychophysical kernels. The upper half presents data of the decision session, the lower half shows the movement session. Purple (fast decision- or tapping-blocks) and green (slow decision- or tapping-blocks) line graphs show how the correlation between momentary evidence and participants’ decisions (y-axis) changes as a function of time (x-axis, in seconds [s]) relative to the response (dashed vertical line). These line graphs depict grand averages across participants, with lighter colors covering 95%- confidence intervals. Vertical colored solid lines represent the expected onset of the decision (phase) per experimental condition, computed as negative average median reaction time across participants. Statistical tests are conducted on the basis of individual slopes from decision onset to peak (as proxy of shifts in decisional speed-accuracy tradeoff), as well as individual latencies of the peak relative to the response (as proxy for sensorimotor delays related to initiating the finger tapping sequence). For individual data, red lines depict participants with a numerically higher value in fast (decision or tapping) blocks as compared to slow blocks, whereas blue lines depict participants with the opposite effect.

As an alternative approach for testing H1 and H2, we also derived so-called psychophysical kernels (PKs) from behavior. As seen in Fig. 3, PKs indicate in how far participant’s decisions relied on momentary decisional evidence, which dynamically changed over the course of the decision phase. PKs show a prototypical steep rise in the correlation between evidence and the participant’s decisions until peaking before the response. This peak marks the point in time at which variations in momentary evidence lead to decisional commitment, after which the corresponding response is prepared. The following steep decline indicates that after this commitment subsequent evidence can no longer influence decisions^53^. Relevant for H1 and H2, properties of PKs are indicative of participant’s behavior: A steeper slope of the PK until its peak is indicative of a shift in the decisional speed-accuracy tradeoff in favor of faster decisions: The steeper slope is the result of variations in momentary evidence being more capable to elicit decisional commitment, which hence tends to occur earlier^53^. In addition, a reduced latency between the peak (as timepoint of decisional commitment) and the subsequent response is indicative of shorter sensorimotor delays related to initiating the finger tapping sequence^53^. For H1, we thus expected a reduced latency of the peak in fast-decision as compared to slow-decision blocks. For H2, we expected a steeper slope in fast-tapping as compared to slow-tapping blocks.

As seen in Fig. 3, we computed PKs after summarizing momentary evidence across timespans of 200, 300, 400 and 500 ms. These timespans reflect possible rates with which momentary evidence may be integrated into decisions. We analyzed data across these timespans since the true rate of evidence integration is unknown. For humans, 200 ms is considered a minimum plausible timespan^44,54,55^. In line with H1, fast-decision blocks were associated with shorter peak latencies than slow-decision blocks (latency difference ≥ 300 ms, all *p*’s ≤ 10^–4^). They were also associated with steeper slopes, consistent with a shift in decisional speed-accuracy tradeoff (slope difference ≥ 0.11 per second, all *p*’s ≤ 0.04), as required by instructions. In line with H2, fast-tapping blocks were associated with steeper slopes than slow-tapping blocks for timespans of 300, 400 and 500 ms (slope difference ≥ 0.07 per second, all *p*’s < 0.05), but not 200 ms (slope difference = 0.10 per second, *p* = 0.30). Tapping requirements did not alter peak latencies (all latency differences = 0, all *p*’s > 0.99), suggesting no change in sensorimotor delays. These results on psychophysical kernels are compatible with behavioral findings, although here sensorimotor delays were directly estimated from PKs, rather than in the separate SRT task, demonstrating the robustness of findings. These results are also consistent with drift–diffusion modelling, which suggested a shift in *decision* *threshold* and *non-decision* *time* in the decision session, as well as a shift in *decision* *threshold* in the movement session.

## Discussion

*Embodied* *decision-making* is a perspective which assumes an inherent functional interdependency between deciding amongst different realizable events and preparing movements corresponding to their implementation^8,34,35^. Previous work remained inconclusive in this regard, since such coregulation was incentivized by task design by requiring both fast decisions and fast movements to obtain rewards under time pressure [e.g. Refs.^17–19^]. Our findings are in support of such interdependency with regards to the pace with which decisions and movements are completed. We hence extend previous work in important ways: Faster as compared to slower decisions were followed by faster finger tapping movements, although such behavior was not encouraged, nor adaptive in the context of the task. Likewise, faster as compared to slower tapping induced faster decision-making, which cannot be easily reconciled with strategic considerations. Taken together, these findings suggest that decisions not only determine movement characteristics, but that properties of movement constrain the time with which decisions are made.

Previous studies suggest that decisions and movements are coregulated to increase capture rate, which declines with the average time and effort required to obtain reward^10^. It is plausible that such coregulation is inherent to the sensorimotor system, and therefore not limited to contexts explicitly promoting such behavior. To address this hypothesis, in the present study coregulating decisions and movements was not effective to alter capture rate: Tapping speed in the movement phase did not change the pacing of a trial, nor did the duration of the decision phase, with the exception of fast-decision blocks, to encourage faster decisions. Hence, it was not adaptive in terms of capture rate to speed up decisions or finger tapping, unless this was explicitly instructed. Importantly, we did not find that understanding of these instructions modulated the experimental effect (see Supplemental Material). Moreover, tapping faster after faster decisions, as observed in the decision session, did not allow to increase the amount of momentary reward earned, as a timeout of three seconds in the movement phase ensured that the tapping sequence was virtually always completed on time (and hence rewarded). Likewise, deciding faster before fast finger tapping, as observed in the movement session, did not allow to increase the amount of momentary reward neither, as faster decisions could not increase probability of success. Finally, changes in effort allocation likely cannot account for these findings neither. As such, one may assume that faster, more effortful finger tapping^56–58^ was compensated by performing shorter, less effortful decisions^59^, and vice versa, explaining the findings in both sessions. However, by nature of the task late decisions benefitted from more conclusive information than early information, which was intentionally held ambiguous or even misleading, to prevent participants from deciding too quickly (see “Materials and methods”). In line with this, *drift* *rate* was significantly reduced in fast-decision blocks as compared to slow-decision blocks, suggesting that deciding with limited evidence and under time pressure may have been more difficult, and hence more effortful, rather than less effortful. In addition, a recent study explicitly tested in a related task whether movement effort is compensated by shortening decision duration, finding no such effect with 31 participants^46^. Given these considerations, we interpret current findings as reflecting inherent properties of the sensorimotor system rather than strategic adaptations with changing task contexts.

If not strategically advantageous, why did the pacing of decisions and movements condition each other? Several brain areas have been proposed, which may take common influence on decision making and movement control. Subcortical structures, specifically the basal ganglia^37–39^ and the locus coeruleus-noradrenergic system^18,19,40^, may broadly regulate activity of cortical sensorimotor areas when time pressure is high. The core idea of these proposals is that neural activity in sensorimotor cortex accumulates towards commitment for a certain motor response^17–19,47^. Under time pressure, these subcortical areas may upregulate neural activity in cortical structures so that such decisional commitment occurs earlier. On the level of cortical premotor and motor areas, such global change in neural activity can be observed as broad disinhibition of the motor system: Beta and mu power, electrophysiological markers of inhibitory control over sensorimotor sites, are reduced when participants are required to report decisions fast through movement^17–19,42^. Likewise, stronger motor disinhibition at corresponding sensorimotor sites is associated with faster response times^7,60,61^ and higher movement velocity [Refs.^62–65^], but see Ref.^66^. Such motor disinhibition may therefore reflect an increased tendency to execute a movement, and hence to report a decision, thereby reducing the time available to decide. Taken together, we propose that subcortical structures, such as basal ganglia or the noradrenergic system may regulate broad changes in neural activity of the sensorimotor cortex when fast action is required, reducing the time taken to decide and to move in a joint fashion. Future studies may turn to electrophysiological correlates of decisions [see Ref.^6^] and movements [see Ref.^7^], to further insight on the cortical mechanisms acting on the level of both decisional and movement control.

Present findings are conceptually compatible with “procedural priming”, which is the generalization of behavior shown in one task to another following task^67,68^. When asked to either ‘choose bananas fast’ or to ‘move the caterpillar fast’, participants may have applied similar strategies to all aspects of the task, leading to observed coregulation effects. Such scenario may seem likely in case participants were unmotivated and hence did not fully pay attention to instructions. Importantly, results presented in the Supplemental Material speak against such concerns. First, overall task performance was high and improved throughout sessions, suggesting that participants were motivated to perform well (see Fig. S3). Secondly, we show that best-performing participants as well as participants who were able to recall instructions correctly after the experiment showed clear coregulation effects. Indeed, these effects were remarkably consistent on an interindividual level (see Fig. 2B,E). Taken together, our data thus suggests that findings were not driven by a subgroup of participants potentially being unmotivated or inattentive to task instructions. That being said, a generalization of behavior across decision and movement phases of the task (i.e., procedural priming) seems likely from an embodied decision-making perspective: If decisions and movements are functionally intertwined and partially rely on shared neural resources, a clear separation of these two aspects of behavior seems difficult^32^. The fact that we observed such coregulation effects, despite the task being designed to discourage generalization, hence strongly suggests that decisions and movements cannot be readily considered functionally distinct. Instead, they may rely on shared neural mechanisms, potentially being susceptible to procedural priming.

At first glance, our findings seem to be at odds with results of Reynaud et al.^24^, who also investigated how movement duration affects decision-making. In contrast to our findings, faster as compared to slower movements were associated with *longer* decisions [see also Ref.^47^]. However, in their task 160 correct decisions completed an experimental block, and faster movements allowed to finish trials faster. Hence, performing slow, accurate decisions and fast movements posed the best strategy to finish early. As such, trials requiring fast movement may have allowed to invest relatively more time in deciding accurately. This was critically different from our experiment where tap duration did not change trial length and where experimental blocks had a fixed duration, independent of performance. As a result, in our study it was not advantageous to trade off decisions and movements, likely leading to the observed coherence of these sensorimotor processes. Bridging findings of both studies, properties of the sensorimotor system may promote a natural matching of the pace of decisions and movements, but contrary policies may be implemented if advantageous in the task context. We propose that the application of contrary policies to decisions and movements may be a later evolutionary accomplishment, relying on additional “cognitive control”-mechanisms^69–71^.

A perspective of embodied decision-making argues against a strictly sequential relationship of decisions and movements. Hence, it is somewhat a caveat that our data analysis was based on approaches assuming that reaction times reflect the additive sum of delays attributable to decision-making and preparing and executing movements. Such assumption was made when subtracting simple reaction times from reaction times to isolate decision durations (see “Materials and methods”), fitting reaction times to drift diffusion models, and estimating sensorimotor delays from psychophysical kernels. Yet, we chose to adopt such approaches to ensure that faster decisions in fast-tapping blocks could not be attributed to shorter sensorimotor delays. A mathematical formalization of behavior from an embodied decision-making perspective is still in its infancy [e.g., Ref.^33^], which will be required in the future to provide an alternative view on sensorimotor behavior.

Our results demonstrate an interplay between decisional and motor processes. Movement characteristics not only depended on properties of decisions preceding them, but surprisingly, they also took distinct influence on when people arrived at their decisions. We interpret these behavioral results in the context of embodied decision-making, whereby shared neural mechanisms may not only enable faster movements but also assist in making decisions in less time. These findings open interesting new perspectives for future research, which may allow to reconceptualize sensorimotor processes and their associated brain regions as a functional gradient ranging from simulating potential action outcomes based on environmental information to establishing preferred action outcomes in the environment through movement.

## Materials and methods

### Participants

The final dataset consisted of 62 right-handed participants (15 male) between 18 and 34 years old (*M* = 23.69, *SD* = 3.29), which were recruited on Facebook in groups for paid participation in academic research at Belgian universities. They affirmed to have normal or corrected-to-normal vision, no colorblindness, no history of neurological, psychiatric or mental disorders and no physical injuries or disabilities. Participants were asked to perform two sessions of the experiment circa seven days apart, of which one was the decision session and the other the movement session; session order was counterbalanced across participants. Data from both sessions was available for 55 out of 62 participants, which were conducted 7.22 days apart on average (range = 5–14 days, *SD* = 1.08). In the cases data was not available for one session (7 participants), the causes were due to early termination of the session, an interruption of the internet connection, a refresh rate of the computer monitor lower than 60 Hz or poor behavioral performance leading to the exclusion of more than 50% of the trials (see “Data analysis”). In sum, data was available for 58 decision sessions (30 as second session) and 59 movement sessions (29 as second session). This was in line with our goal to reach 60 participants per session, which was set a priori due to practical limitations. A sensitivity power analysis conducted in G* *Power* *3.1*^72^ indicated that 60 participants would allow to detect small-to-medium effect sizes (*d*_*z*_ ≥ 0.37) in paired-samples *t*-tests, and small-to-medium correlations (*r*_*s*_ ≥ 0.25) with 80% power, which we considered satisfactory. Although the signed-rank tests reported in this manuscript may possess slightly less power than *t*-tests, the computed minimum detectable effect size of *d*_*z*_ ≥ 0.37 may still be indicative of the power achieved in this study, as similar statistical methods for nonparametric tests are not readily available. Apart from these 62 participants in the final dataset, eight more participants started at least one session, but were excluded due to similar reasons as stated above.

Paid 10 Euro per hour, participants received 25 Euro for circa 2.5 h split into two experimental sessions, plus a performance-dependent bonus (see “Experimental task”), which was circa 5 Euro. The protocol was approved by the institutional review board of the Catholic University of Louvain, Brussels, Belgium, and was in compliance with the principles of the Declaration of Helsinki. Written, informed consent was given by emailing photographed and hand-signed consent forms to the experimenter.

### Experimental task

As seen in Fig. 1, a trial started with a light gray fixation dot on a dark gray background, shown for 100–500 ms. In the subsequent pre-decision phase, two ‘baby bananas’ appeared on the left and right of the fixation dot at a 30-degree angle and covered a circular track of 60 degrees, crossing each other, to arrive centrally below the fixation dot, separated by a horizontal gap. With a fixed duration of 1000 ms, this pre-decision phase telegraphed the onset of the decision phase. In this next phase, bananas grew progressively, with their tips following a circular track around the fixation dot, whereas their ends remained in place. With every refresh of the screen (i.e., at a rate of 60 Hz), pseudo-randomly either the left or right banana was extended by 2°, creating the illusion of continuous growth at a non-constant speed. If uninterrupted by the participant’s response, the bananas kept growing for 179 frames or 2983 ms (hereafter: 3000 ms) to cover 358° around the fixation dot. An uneven number of frames ensured that one banana was always longer by the end of a trial. Participants had to select the anticipated longer banana before the end of the decision phase by pressing the S-key (left banana) or L-key (right banana) with their respective index finger. With this first finger tap, the movement phase was initiated by placing a rose caterpillar below the central fixation dot on a circular track parallel to the one of the chosen banana, which itself faded into the background within 200 ms. The caterpillar extended with each key press, including the initial decision, by 36 degrees along the circular track, with movement animations lasting circa 80 ms each (5 frames). In addition to the initial decision, participants thus had to tap four more times to move the caterpillar head to a position above the fixation dot. Important for the purpose of the study, the movement phase had a fixed duration of 3000 ms. As such, the subsequent phase did not begin earlier even if participants completed the movements earlier [cf. ^27^]. To represent this fixed duration of the movement phase visually, the fixation dot disappeared with phase onset and gradually returned to initial opacity by phase offset. In addition to the caterpillar which remained onscreen, bananas additionally reappeared within the last 200 ms of the movement phase. If participants had not responded within 3000 ms during the decision phase earlier, the caterpillar did not appear, but participants still had to sit out the movement phase. Next, as a post-decision phase, bananas completed their growth paths in a similar fashion as the decision phase until they covered an approximately full circle around the fixation dot. In this way, for all experimental conditions except one (see the following paragraph), bananas grew for a fixed duration of 3000 ms on every trial independent of the timing of participant’s decision. In a feedback phase lasting 800 ms, the outline of the chosen banana was highlighted in either green or red, depending on the correctness of their decision. If participants had not responded within the specified time of either the decision or movement phase, the fixation dot was replaced with the caption ‘decide faster!’ or ‘tap faster!’, respectively. A final intertrial phase of 700–1000 ms followed, which was visually identical to the fixation phase.

Trials were embedded in different blocks, which manipulated either decision speed or tapping speed experimentally, depending on the session: In the decision session, blocks encouraged either fast decisions or slow (accurate) decisions, whereas in the movement session, either fast or slow finger tapping was required. Blocks in the decision session differed in that fast-decision blocks exceptionally allowed to shorten the duration of a trial by deciding faster: In contrast to all other conditions (see Fig. 1), bananas grew four times faster in the post-decision phase (by only showing every fourth frame of growing bananas). Additionally, fast-decision blocks were not limited to 40 trials as in other blocks, but instead the length of these blocks was fixed to 5 min and 30 s. Participants were instructed to select as many bananas as possible and were informed that choosing bananas quickly would allow them to complete more trials. To encourage this behavior, a block-based bonus of 10 cents was given when participants ‘completed more rounds than average’ (i.e., completing at least 40 trials in fast-decision blocks). In contrast, for slow-decision blocks, participants were informed that exactly 40 trials had to be completed, with a fixed trial duration independent of behavior. Here, participants had to select the correct banana as often as possible. This was again encouraged by rewarding an ‘above average’ amount of correctly chosen bananas with a block-based bonus of 10 cents (i.e., when at least 85% of the decisions were correct). Importantly, it was stressed that tapping speed in the movement phase would not alter trial duration in any experimental condition. This fact was further confirmed with the visual representation of an example trial, which showed that deciding faster in fast-decision blocks was the only circumstance in which behavior would change trial duration.

Blocks in the movement session differed in that fast-tapping blocks required to move the caterpillar with a minimum speed, whereas slow-tapping blocks were limited by maximum speed. For this purpose, a snake either chased the caterpillar during the movement phase or retreated from it, both at a constant speed calibrated individually per participant (see Session Protocol). In fast-tapping blocks, the snake was initially placed 36 degrees behind the caterpillar and moved at a uniform velocity corresponding to 85% of maximum tapping speed. In slow-tapping blocks, the snake was initially placed 36 degrees in front of the caterpillar and retreated with a constant pace corresponding to 59.5% of maximum tapping speed. Depending on the condition, the snake’s color was either purple or green, counterbalanced between participants. If participants tapped too slowly or too quickly, respectively, the caterpillars head would coincide with the snake’s head and both animals would stop moving. In this case, novel responses would no longer be registered, otherwise the trial continued as described earlier. Also for the movement session an example trial demonstrated that trial duration was fixed and thus independent of the participants’ behavior.

In each session, participants performed four times each of the two block types: Eight blocks of (at least) 40 trials presented in randomized order resulted in (at least) 160 trials per experimental condition. Each block started with a fixed instruction screen of at least 20 s before moving on, ensuring that instructions for the following block could not be skipped. Each first trial of a block had an additional 700 ms of fixation. To further remind participants of the current instructions in each block, a symbol representing each of the four block types was shown in the upper right corner of the screen throughout (see Fig. 1). To keep participants engaged throughout the task, 1 point could be earned for each correctly performed decision and movement, respectively (i.e., a maximum of 2 points per trial). After each block, the total number of accumulated points for correct decisions and for correct movements were converted to monetary bonuses at a rate of 0.4 cent per point, in addition to feedback on the possible block-based bonus of 10 cent in decision sessions. Between blocks, participants were further informed about the number of remaining blocks of the current session.

Trial difficulty was controlled between experimental conditions by adjusting the rate at which the winning banana outgrew the other over time: a more asymmetric growth results in an easier decision. We intermixed four trial types which were chosen in analogy to similar approaches in the literature^22,24,39,45,47,48^: In ‘obvious’ trials, one banana grew larger than the other one early on and remained so for the remainder of the trial. In ‘ambiguous’ trials, bananas remained in competition for length for about halfway of the trial, before one banana proceeded to take the lead. In ‘misleading’ trials, one banana seemed to outgrow the other early on in the trial but lost that competition by the end of the trial. Finally, in ‘random’ trials bananas could show any growing pattern. Each experimental condition consisted of 30% obvious, 30% ambiguous, 20% misleading and 20% random trials.

### Session outline

The online experiment was built in lab.js, hosted on Open-Lab^73^, and was accessed via the Google Chrome browser. It required a screen of at least 15 inch in diameter with a refresh rate of 60 Hz, and was run in full screen mode. At the start of each session, participants were asked to create a calm environment by using a solitary room and by removing possible sources of noise. An overview of the session outline can be seen in Supplemental Fig. S4.

The movement session always started with a so-called ‘keyboard calibration’, which was skipped for the decision session. This segment allowed to estimate maximum tapping speed, although this goal was not disclosed. Within four-second intervals, the S- or L-key had to be tapped repeatedly as fast as possible. Each trial consisted of a countdown represented as a pie chart, which declined from 360° (full circle) to 0° (no circle) within four seconds. The letter which had to be tapped was superimposed. A counter for the total number of key taps was shown below the pie chart, both were reset with each new trial. After each trial, the feedback to tap ‘FASTER!’ was given irrespective of performance. Participants performed eight trials per key (the beginning key was randomized) and the maximum tapping speed was calculated as average tapping speed for the last six trials of both keys.

The first out of two sessions continued with a familiarization segment. This segment introduced the decisional aspect of the task through visual animations and written text. Instructions could be repeated at will by navigating through instruction screens with dedicated keys, but new instructions could not be skipped. A simpler version of the task with only the decision phase could then be practiced in 20 trials, where a trial required only one key press to report the decision. Practice had to be completed up to three times if the proportion of correct decisions did not reach at least 70%. In that case, participants were given the choice to re-read or skip instructions before repeating practice trials. All sessions then continued with a training phase, which introduced the movement phase of the trial. Participants were then informed about the two experimental blocks in the session. This full task had to be practiced in another 20 trials, with 10 trials in each condition. Training had to be again completed up three times if either a decision accuracy of at least 70% was not reached, or the required number of key taps was below 70%.

The main experiment followed with circa 320 trials. Participant’s understanding of the instructions was afterward probed with a questionnaire: Statements on the influence of behavior on trial duration had to be rated on agreement (see Table S2). Participants with a good task understanding should indicate that choosing a banana faster or moving the caterpillar faster does not alter trial duration, except for fast decisions in fast-decision blocks.

Finally, participants performed a simple reaction time (SRT) task, which was comparable to the main task, but stripped of decisional aspects by giving away the correct response in advance. This task allowed to estimate sensorimotor delays attributable to pressing the first key of a tapping sequence (see “Data analysis”, Fig. S5 and Supplemental Material). In the pre-decision phase of the SRT task, one motionless baby banana was pointing towards the left or right, randomized between trials. After a delay of 1000–1600 ms, a fully extended banana covered 180° in the same direction. As soon as the banana thus fully grew, the corresponding key had to be tapped. Premature responses were followed with the words ‘too early!’ for three seconds instead of the extended banana. All other phases of the trial were identical to the main task, with the exception that there was no post-decision phase. The movement session entailed 40 SRT trials requiring fast tapping (promoted by a chasing snake) and 40 SRT trials requiring slow tapping (promoted by a retreating snake; with block order randomized). The decision session entailed 40 SRT trials with no restrictions on tapping speed (with no snake), as was the case for the main task.

### Data analysis

#### Behavior

Endpoint measures were computed which reflect decision and motor performance: *Decision* *duration* was computed for each participant as average reaction times in each experimental condition minus the average reaction time in the corresponding SRT block [cf. Refs.^22,24,46,47,55,57,71^]. This ensured that only the time taken to decide was compared between experimental blocks, without confounding it with the time needed to perform a first finger tap. *Success* *probability* reflects the objective probability that the decision is correct, given the length difference in bananas at the time of the decision (see Supplemental Material for further details on this measure). *Tap* *duration* was measured as average time difference between successive finger taps and hence reflects how fast participants moved the caterpillar in the movement phase of the task. *Movement* *accuracy* reflects the proportion of trials in which the four finger taps were carried out at the proper pace in the movement phase.

Trials with no response during the decision phase were removed from both the main experiment and SRT task. Trials with anticipation errors were also excluded: These regarded key presses occurring in the pre-decision phase, decisions with a duration less than 150 ms in case of the main task, or with a reaction time less than 50 ms in case of the SRT task. Trials of the main experiment were excluded if frame rate deviated by at least 1 Hz from the desired 60 Hz in either pre-decision, decision or movement phases. For the SRT task frame rate was not recorded. *Decision* *duration* and *success* *probability* were calculated across trials with correct movements (i.e., the four finger taps were carried out at the proper pace within the movement phase). *Movement* *accuracy* was calculated based on trials with correct decisions. Overall, 8% (decision duration and success probability) or 20% (movement accuracy) of trials were thus removed from the main experiment, and 7% of trials were removed from the SRT task.

Statistical tests were conducted separately for each session, which allowed more targeted testing of the hypotheses. Robust statistical methods were used to address apparent issues with non-normality and heteroscedasticity (see Fig. 2): To compare two measurements of the same participants, signed-rank tests were used. Monotonic relationships were tested using Spearman’s rank correlations. Due to evident bivariate outliers to which these correlations are sensitive (see Fig. 2F), correlations were further tested for robustness by repeating same tests after removing outliers^74^. Outliers were detected by the fast minimum covariance determinant estimator-algorithm^74^. Statistical analysis was conducted in R^75^, relying on packages BDSA, stats and rrcov.

#### Drift diffusion modelling

To corroborate the interpretation of behavioral data as reflecting shifts in duration with which decisions and finger tap movements are performed, drift diffusion models were fit to reaction times in the decision phase of the task with the hddm- package in Python (version 0.7.3)^76^. Four parameters characterize different aspects of the sensorimotor process: The *decision* *threshold* reflects the decisional speed-accuracy tradeoff with higher values indicating that more certainty is needed before commitment, resulting in slower but more accurate decisions. The *drift* *rate* reflects the efficiency with which a correct decision can be made. It therefore increases with lower task difficulty, and higher motivation and performance. The *non-decision* *time* represents sensorimotor delays unrelated to decision-making, such as sensation and motor processes for preparing and executing the first finger tap used for reporting the decision. Although the *non-decision* *time* also typically captures changes in sensation, the assumption that changes in *non-decision* *times* are primarily driven by motor delays seems reasonable given previous studies attesting that motor delays are a major contributor to overall reaction times and are likely to change with task contexts^10,21,22^. The *response* *bias* reflects an a-priori tendency to prefer one response over the other, for example when one response is more rewarded than the other.

In line with our hypothesis, for each session a model allowing the *decision* *threshold* and *non-decision* *time* to change with experimental conditions was fit. This model was compared with models of different complexity (see Table S1). The most complex model assumed that, in addition to the *decision* *threshold* and *non-decision* *time*, also the *drift* *rate* differed between experimental conditions. All other models assumed a fixed *drift* *rate*, as well as at least one of the parameters of interest (*decision* *threshold* and *non-decision* *time*) to be fixed between experimental conditions. For all fitted models, we assumed no *response* *bias* since an equal amount of left and rightward responses was required in the task (i.e., the *response* *bias* was assumed to be 0.5, see, e.g., Refs.^38,77^). Although this may be a simplifying assumption, it allows for a more powerful test of the effects of interest^78,79^. Moreover, our behavioral findings likely cannot be accounted for by *response* *biases*. For each model, ‘flexible’ parameters were modelled as within-subject effects by fitting one parameter value to the slow condition of the respective session, and another parameter value reflected the deviation thereof in the fast condition (see Table S1). Models were hierarchical in that parameter values of each subject were assumed to spread around the group mean of that parameter. Correct and incorrect responses were taken as upper and lower boundaries of the drift diffusion process. Five percent of responses were assumed to be outliers, as recommended to improve model fit^76^.

With this approach, modelling provided an alternative way of separating shifts in decisional speed-accuracy tradeoff from sensorimotor delays and hence testing H1 and H2, without the SRT task, to ensure that presented findings are consistent across different methodological approaches. Consistent with this aim, reaction times less than 150 ms were discarded as anticipation errors, rather than decision durations below 150 ms as was the case in the behavioral analysis. Each model in Table S1 was fit twice with 5000 discarded samples as burn-in and keeping every third of 6000 subsequent samples, resulting in 4000 final posterior samples per parameter. Model convergence was assessed by visual inspection of the Markov-chain traces, as well as computation of the Gelman-Rubin statistic. With values below 1.1 considered satisfactory by convention^80^, Gelman-Rubin values indicated convergence for all models of the decision session (all below 1.04), as well as the movement session (all below 1.01). Models were then compared based on the bias-corrected Bayesian Predictive Information Criterion (BPIC)^50,51^, since this criterion does not require the true model to be amongst candidate models. Best-fitting models were further ratified by posterior predictive checks: New data was simulated from best-fitting models and visually compared to the real empirical data (see Fig. S6), suggesting good agreement between model predictions and reality. As a final step in this model analysis, for the best-fitting models, the probability of the existence of within-subject effects was expressed as proportion of posterior samples smaller than zero for the respective parameter. This proportion reflects the estimated probability that parameter values are more negative in fast conditions than in slow conditions. In contrast to *p*-values, a probability exceeding 97% indicates that an effect likely exists^81^.

#### Psychophysical kernels

To further test H1 and H2 in an alternative fashion, which does not rely on the SRT task (behavioral analysis) or behavioral modelling (drift diffusion modelling) to estimate sensorimotor delays, we computed psychophysical kernels. These kernels outline in how far newly presented momentary evidence is predictive of the subsequent decision, depending on when this evidence is presented relative to the response. After discarding reaction times less than 150 ms, as a first step in computing these kernels, momentary evidence presented throughout a trial was quantified: In representative timespans of 200, 300, 400 or 500 ms, screen refreshes (frames) shifting evidence in favor of either the left (− 1) or right (+ 1) banana were summed up to a total value. As such, momentary evidence could either be positive or negative, with more extreme values indicating stronger momentary evidence towards the left or right. Momentary evidence was summarized in this way by moving the respective timespan one frame at a time across the entire sequence of decisional evidence presented throughout a trial. Momentary evidence and decisions (− 1 for left and + 1 for right responses) were then correlated across trials for each participant, experimental condition and time window of presentation (see Ref.^82^, for a similar approach). As seen in Fig. 3, resulting point-biserial correlations characterize in how far momentary evidence predicts decisions, depending on the time of presentation relative to the response. To assess shifts in decisional speed-accuracy tradeoff, for each participant and experimental condition, the slopes of psychophysical kernels were extracted from the expected decision onset (i.e., negative median reaction time) to the expected decision offset (i.e., peak of the kernel, as seen in Fig. 3, see Refs.^43,83^, for similar approaches). Steeper slopes suggest a shift in decisional speed-accuracy tradeoff in favor of faster decisions, since the slope is indicative of the propensity with which momentary evidence may elicit decisional commitment^53^. To assess shifts in sensorimotor delays related to initiating the finger tapping sequence, for each participant and experimental condition, the latency between the peak and subsequent response was computed. A shorter latency suggests faster sensorimotor delays^53^. We compared slopes and peak latencies for each experimental session with signed-rank tests, or with paired *t*-tests in case Shapiro–Wilk tests were not violated (see Refs.^83,84^, for similar approaches). Testing across multiple timespans was corrected by the method of Holm for each session^85^.

### Significance statement

*Embodied* *decision-making* assumes functional interdependency between deciding amongst realizable events and their implementation through movement. In a task where coregulating decisions and movements was not promoted, we show that faster movements induce faster decisions and vice versa. Results are further supported by drift diffusion modelling and inspection of psychophysical kernels. These findings suggest that decisions determine movement characteristics, but also that movement characteristics constrain the duration of decisions. This opens interesting perspectives on sensorimotor processes and associated brain regions, suggesting a functional gradient ranging from evaluating potential action outcomes based on sensory information to establishing preferred outcomes through movement.

## Supplementary Information

Supplementary Information.

## Data Availability

All data and accompanying scripts are available on the Open Science framework for free access: https://osf.io/dk7a2/.

## References

[1] Ratcliff R, McKoon G (2008). The diffusion decision model: Theory and data for two-choice decision tasks. Neural Comput..

[2] Sternberg S (1969). Memory-scanning: Mental processes revealed by reaction-time experiments. Am. Sci..

[3] Farrar DC, Mian AZ, Budson AE, Moss MB, Killiany RJ (2018). Functional brain networks involved in decision-making under certain and uncertain conditions. Neuroradiology.

[4] Welniarz Q (2022). Identification of a brain network underlying the execution of freely chosen movements. Cereb. Cortex.

[5] Desender K, Ridderinkhof KR, Murphy PR (2021). Understanding neural signals of post-decisional performance monitoring: An integrative review. Elife.

[6] Kelly SP, O’Connell RG (2015). The neural processes underlying perceptual decision making in humans: Recent progress and future directions. J. Physiol. Paris.

[7] Wessel JR (2020). β-bursts reveal the trial-to-trial dynamics of movement initiation and cancellation. J. Neurosci..

[8] Cisek P, Pastor-Bernier A (2014). On the challenges and mechanisms of embodied decisions. Philos. Trans. R. Soc. B Biol. Sci..

[9] Klein-Flügge MC, Bestmann S (2012). Time-dependent changes in human corticospinal excitability reveal value-based competition for action during decision processing. J. Neurosci..

[10] Shadmehr R, Reppert TR, Summerside EM, Yoon T, Ahmed AA (2019). Movement vigor as a reflection of subjective economic utility. Trends Neurosci..

[11] Calderon CB, Van Opstal F, Peigneux P, Verguts T, Gevers W (2018). Task-relevant information modulates primary motor cortex activity before movement onset. Front. Hum. Neurosci..

[12] de Lange FP, Rahnev DA, Donner TH, Lau H (2013). Prestimulus oscillatory activity over motor cortex reflects perceptual expectations. J. Neurosci..

[13] Klein P-A, Olivier E, Duque J (2012). Influence of reward on corticospinal excitability during movement preparation. J. Neurosci..

[14] Derosiere G (2018). Visuomotor correlates of conflict expectation in the context of motor decisions. J. Neurosci..

[15] Duque J, Petitjean C, Swinnen SP (2016). Effect of aging on motor inhibition during action preparation under sensory conflict. Front. Aging Neurosci..

[16] Derosiere G, Thura D, Cisek P, Duque J (2022). Hasty sensorimotor decisions rely on an overlap of broad and selective changes in motor activity. PLoS Biol..

[17] Kelly SP, Corbett EA, O’Connell RG (2021). Neurocomputational mechanisms of prior-informed perceptual decision-making in humans. Nat. Hum. Behav..

[18] Murphy PR, Boonstra E, Nieuwenhuis S (2016). Global gain modulation generates time-dependent urgency during perceptual choice in humans. Nat. Commun..

[19] Steinemann NA, O’Connell RG, Kelly SP (2018). Decisions are expedited through multiple neural adjustments spanning the sensorimotor hierarchy. Nat. Commun..

[20] Freeman JB (2018). Doing psychological science by hand. Curr. Dir. Psychol. Sci..

[21] Spieser L, Servant M, Hasbroucq T, Burle B (2017). Beyond decision! Motor contribution to speed–accuracy trade-off in decision-making. Psychon. Bull. Rev..

[22] Thura D (2020). Decision urgency invigorates movement in humans. Behav. Brain Res..

[23] Burk D, Ingram JN, Franklin DW, Shadlen MN, Wolpert DM (2014). Motor effort alters changes of mind in sensorimotor decision making. PLoS ONE.

[24] Reynaud AJ, Saleri Lunazzi C, Thura D (2020). Humans sacrifice decision-making for action execution when a demanding control of movement is required. J. Neurophysiol..

[25] Hagura N, Haggard P, Diedrichsen J (2017). Perceptual decisions are biased by the cost to act. Elife.

[26] Marcos E, Cos I, Girard B, Verschure PF (2015). Motor cost influences perceptual decisions. PLoS ONE.

[27] Morel P, Ulbrich P, Gail A (2017). What makes a reach movement effortful? Physical effort discounting supports common minimization principles in decision making and motor control. PLoS Biol..

[28] Barsalou LW (2008). Grounded cognition. Annu. Rev. Psychol..

[29] Cos I, Duque J, Cisek P (2014). Rapid prediction of biomechanical costs during action decisions. J. Neurophysiol..

[30] Shadmehr R, Huang HJ, Ahmed AA (2016). A representation of effort in decision-making and motor control. Curr. Biol..

[31] Yoon T, Geary RB, Ahmed AA, Shadmehr R (2018). Control of movement vigor and decision making during foraging. Proc. Natl. Acad. Sci. U. S. A..

[32] Cisek P (2019). Resynthesizing behavior through phylogenetic refinement. Atten. Percept. Psychophys..

[33] Lepora NF, Pezzulo G (2015). Embodied choice: How action influences perceptual decision making. PLoS Comput. Biol..

[34] Yoo SBM, Hayden BY (2018). Economic choice as an untangling of options into actions. Neuron.

[35] Wispinski, N. J., Gallivan, J. P. & Chapman, C. S. Models, movements, and minds: Bridging the gap between decision making and action. *Ann.**N.**Y.**Acad.**Sci.* 1–22 (2018). 10.1111/nyas.13973 30312476

[36] Cisek P (2007). Cortical mechanisms of action selection: The affordance competition hypothesis. Philos. Trans. R. Soc. B Biol. Sci..

[37] Forstmann BU (2008). Striatum and pre-SMA facilitate decision-making under time pressure. Proc. Natl. Acad. Sci..

[38] Herz DM, Zavala BA, Bogacz R, Brown P (2016). Neural correlates of decision thresholds in the human subthalamic nucleus. Curr. Biol..

[39] Thura D, Cisek P (2017). The basal ganglia do not select reach targets but control the urgency of commitment. Neuron.

[40] Hauser TU, Moutoussis M, Purg N, Dayan P, Dolan RJ (2018). Beta-blocker propranolol modulates decision urgency during sequential information gathering. J. Neurosci..

[41] Jaśkowski P, van der Lubbe RH, Wauschkuhn B, Wascher E, Verleger R (2000). The influence of time pressure and cue validity on response force in an S1–S2 paradigm. Acta Physiol. (Oxf).

[42] Pastötter B, Berchtold F, Bäuml KT (2012). Oscillatory correlates of controlled speed-accuracy tradeoff in a response-conflict task. Hum. Brain Mapp..

[43] Spieser, L., Kohl, C., Forster, B., Bestmann, S. & Yarrow, K. Neurodynamic evidence supports a forced-excursion model of decision-making under speed/accuracy instructions. *eNeuro* **5**, (2018). 10.1523/ENEURO.0159-18.2018 PMC6019391 29951578

[44] Cisek P, Puskas GA, El-Murr S (2009). Decisions in changing conditions: The urgency-gating model. J. Neurosci..

[45] Derosiere G, Thura D, Cisek P, Duque J (2019). Motor cortex disruption delays motor processes but not deliberation about action choices. J. Neurophysiol..

[46] Saleri Lunazzi, C., Reynaud, A. J. & Thura, D. Dissociating the impact of movement time and energy costs on decision-making and action initiation in humans. *Front.**Hum.**Neurosci.* **15**, (2021). 10.3389/fnhum.2021.715212 PMC8592235 34790104

[47] Thura D, Cisek P (2016). Modulation of premotor and primary motor cortical activity during volitional adjustments of speed-accuracy trade-offs. J. Neurosci..

[48] Thura D, Cisek P (2014). Deliberation and commitment in the premotor and primary motor cortex during dynamic decision making. Neuron.

[49] Cousineau D (2005). Confidence intervals in within-subject designs: A simpler solution to Loftus and Masson’s method. Tutor. Quant. Methods Psychol..

[50] Ando T (2007). Bayesian predictive information criterion for the evaluation of hierarchical Bayesian and empirical Bayes models. Biometrika.

[51] Heathcote, A., Brown, S. D. & Wagenmakers, E.-J. An introduction to good practices in cognitive modeling. In *An**Introduction**to**Model-Based**Cognitive**Neuroscience* 25–48 (Springer, 2015).

[52] Rae B, Heathcote A, Donkin C, Averell L, Brown S (2014). The hare and the tortoise: Emphasizing speed can change the evidence used to make decisions. J. Exp. Psychol. Learn. Mem. Cogn..

[53] Okazawa G, Sha L, Purcell BA, Kiani R (2018). Psychophysical reverse correlation reflects both sensory and decision-making processes. Nat. Commun..

[54] Carland MA, Thura D, Cisek P (2015). The urgency-gating model can explain the effects of early evidence. Psychon. Bull. Rev..

[55] Scott SH (2016). A functional taxonomy of bottom-up sensory feedback processing for motor actions. Trends Neurosci..

[56] Lutz K, Koeneke S, Wüstenberg T, Jäncke L (2004). Asymmetry of cortical activation during maximum and convenient tapping speed. Neurosci. Lett..

[57] Arias P (2015). Central fatigue induced by short-lasting finger tapping and isometric tasks: A study of silent periods evoked at spinal and supraspinal levels. Neuroscience.

[58] Jäncke L, Lutz K, Koeneke S (2006). Converging evidence of ERD/ERS and BOLD responses in motor control research. Prog. Brain Res..

[59] Petitet, P., Attaallah, B., Manohar, S. G. & Husain, M. The computational cost of active information sampling before decision-making under uncertainty. *Nat.**Hum.**Behav.* 1–12 (2021). 10.1038/s41562-021-01116-6 34045719

[60] Little S, Bonaiuto J, Barnes G, Bestmann S (2019). Human motor cortical beta bursts relate to movement planning and response errors. PLoS Biol..

[61] Meziane HB (2015). Movement preparation and bilateral modulation of beta activity in aging and Parkinson’s disease. PLoS ONE.

[62] Chen CC (2007). Excessive synchronization of basal ganglia neurons at 20 Hz slows movement in Parkinson’s disease. Exp. Neurol..

[63] Chen CC (2011). Stimulation of the subthalamic region at 20 Hz slows the development of grip force in Parkinson’s disease. Exp. Neurol..

[64] Pogosyan A, Gaynor LD, Eusebio A, Brown P (2009). Boosting cortical activity at beta-band frequencies slows movement in humans. Curr. Biol..

[65] Torrecillos F (2018). Modulation of beta bursts in the subthalamic nucleus predicts motor performance. J. Neurosci..

[66] Tatti E (2019). Beta modulation depth is not linked to movement features. Front. Behav. Neurosci..

[67] Eriksson K, Jansson F (2016). Procedural priming of a numerical cognitive illusion. Judgm. Decis. Mak..

[68] Förster J, Liberman N, Friedman RS (2007). Seven principles of goal activation: A systematic approach to distinguishing goal priming from priming of non-goal constructs. Pers. Soc. Psychol. Rev..

[69] Botvinick MM, Braver TS (2015). Motivation and cognitive control: From behavior to neural mechanism. Annu. Rev. Psychol..

[70] Kouneiher F, Charron S, Koechlin E (2009). Motivation and cognitive control in the human prefrontal cortex. Nat. Neurosci..

[71] Shenhav A, Botvinick MM, Cohen JD (2013). The expected value of control: An integrative theory of anterior cingulate cortex function. Neuron.

[72] Faul F, Erdfelder E, Buchner A, Lang A-G (2009). Statistical power analyses using G* Power 3.1: Tests for correlation and regression analyses. Behav. Res. Methods.

[73] Henninger F, Shevchenko Y, Mertens UK, Kieslich PJ, Hilbig BE (2021). lab.js: A free, open, online study builder. Behav. Res. Methods.

[74] Rousselet GA, Pernet CR (2012). Improving standards in brain-behavior correlation analyses. Front. Hum. Neurosci..

[75] R Core Team. *R:**A**Language**and**Environment**for**Statistical**Computing* (R Foundation for Statistical Computing, Austria, 2015, 2018).

[76] Wiecki TV, Sofer I, Frank MJ (2013). HDDM: Hierarchical Bayesian estimation of the drift-diffusion model in Python. Front. Neuroinform..

[77] Assink, N. *et**al.* Does time pressure induce tunnel vision? An examination with the Eriksen Flanker Task by applying the Hierarchical Drift Diffusion Model. In *Proceedings**of**the**International**Conference**on**Neural**Networks–Fuzzy**Systems**(NN-FS**2015)* 30–40 (2015).

[78] Lerche V, Voss A (2016). Model complexity in diffusion modeling: Benefits of making the model more parsimonious. Front. Psychol..

[79] van Ravenzwaaij D, Donkin C, Vandekerckhove J (2017). The EZ diffusion model provides a powerful test of simple empirical effects. Psychon. Bull. Rev..

[80] Brooks SP, Gelman A (1998). General methods for monitoring convergence of iterative simulations. J. Comput. Graph. Stat..

[81] Makowski D, Ben-Shachar MS, Chen S, Lüdecke D (2019). Indices of effect existence and significance in the Bayesian framework. Front. Psychol..

[82] Bitzer, S., Park, H., Maess, B., von Kriegstein, K. & Kiebel, S. J. Representation of perceptual evidence in the human brain assessed by fast, within-trial dynamic stimuli. *Front.**Hum.**Neurosci.* **14**, (2020). 10.3389/fnhum.2020.00009 PMC7010639 32116600

[83] Levi, A. J., Yates, J. L., Huk, A. C. & Katz, L. N. Strategic and dynamic temporal weighting for perceptual decisions in humans and macaques. *Eneuro* **5**, (2018). 10.1523/ENEURO.0169-18.2018 PMC6220584 30406190

[84] Hubert-Wallander B, Boynton GM (2015). Not all summary statistics are made equal: Evidence from extracting summaries across time. J. Vis..

[85] Chen S-Y, Feng Z, Yi X (2017). A general introduction to adjustment for multiple comparisons. J. Thorac. Dis..

