# Supplementary material for: Movement characteristics impact decision-making and vice versa
Source: Sci Rep. 2023 Feb 25;13:3281. doi: 10.1038/s41598-023-30325-4 (PMC9968293; doi:10.1038/s41598-023-30325-4)
Supplement: Supplementary file 1 — Supplementary Information. [file 41598_2023_30325_MOESM1_ESM.pdf]

SUPPLEMENTAL MATERIAL

Title: Movement characteristics impact decision-making and vice versa

Brief title: Movements impact decisions and vice versa

Authors: Thomas Carsten*, Fanny Fievez, Julie Duque

***Corresponding author:** Thomas Carsten, thomas.carsten@uclouvain.be, https://orcid.org/0000-0002-6651-4958

Affiliation of all authors: CoActions Lab, Institute of Neuroscience, Université catholique Louvain, Tour Bernard Tower, 2d floor, Ave Hippocrate 54, 1200 Bruxelles, Belgium

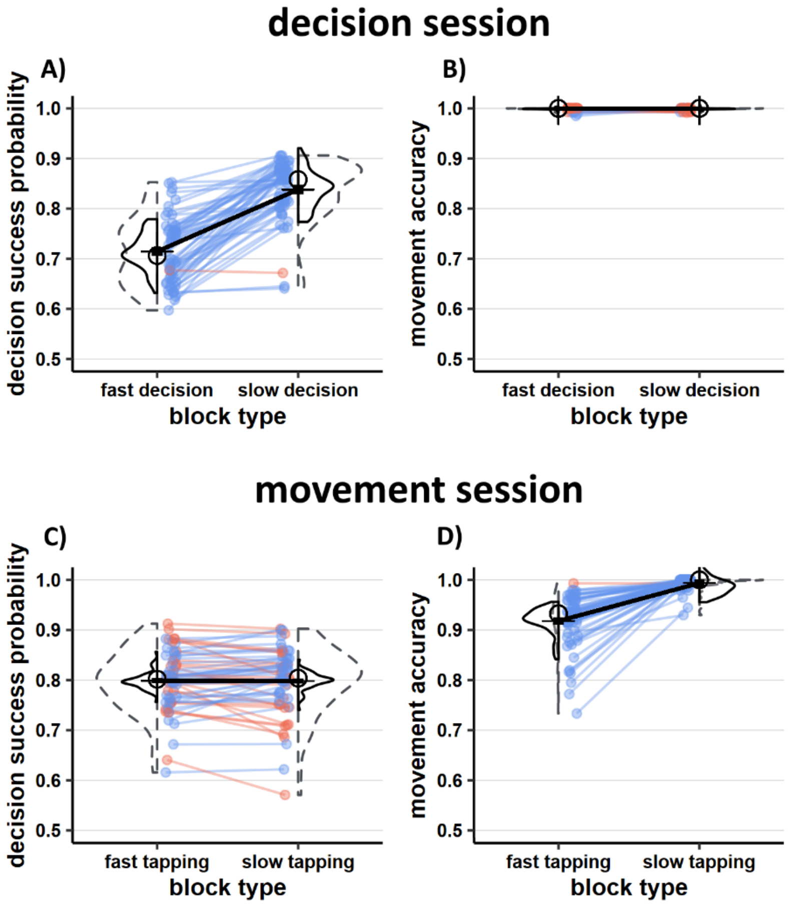

Figure S1. *Supplemental behavioral results.* **A)** Probability that decisions led to successful outcomes was significantly decreased in fast-decision as compared to slow-decision blocks. **B)** In the decision session, participants absolved virtually all tapping movements correctly, with no significant difference between blocks. **C)** There was no significant change in decisional success probability from slow-tapping to fast-tapping blocks. **D)** Participants performed significant less fast-tapping movements correctly than slow-tapping movements.

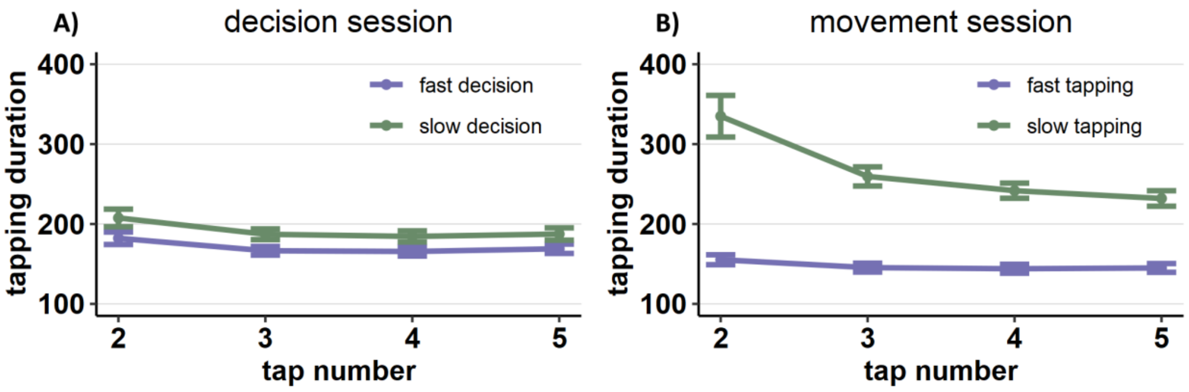

Figure S2. Average duration per finger tap in the movement phase for each experimental condition. **A)** In the decision session, each tap in the movement phase was faster when fast decisions as compared to slow decisions were required, demonstrating the anticipated experimental effect. **B)** In the movement session, each tap in the movement phase was faster when fast tapping as compared to slow decisions were required, demonstrating that the experimental manipulation was successful. Note that the first finger tap in each trial reflected the reporting of the decision in the decision phase, hence for this analysis 2nd to 5th finger tap are considered. Error bars reflect 95%- confidence intervals.

Table S1. *Model fit of drift diffusion models.*

	BPIC	
flexible parameters	decision session	movement session
a, v, t	49012*	45768
a, t	49814	45763*
a	52361	46784
t	51947	45858
none	58146	47020

Note. Bayesian Predictive Information Criterion (BPIC) scores for five different drift diffusion models, which varied in model complexity. Lower values indicate better model fit. Best fitting models are marked with an asterisk. Note that BPIC scores should not be directly compared between sessions, since models were fit to different data. Flexible parameters were those which were allowed to differ between experimental conditions. a = decision threshold, v = drift rate, t = non-decision time.

Excluding lack of motivation or attention as potential drivers for coregulation effects

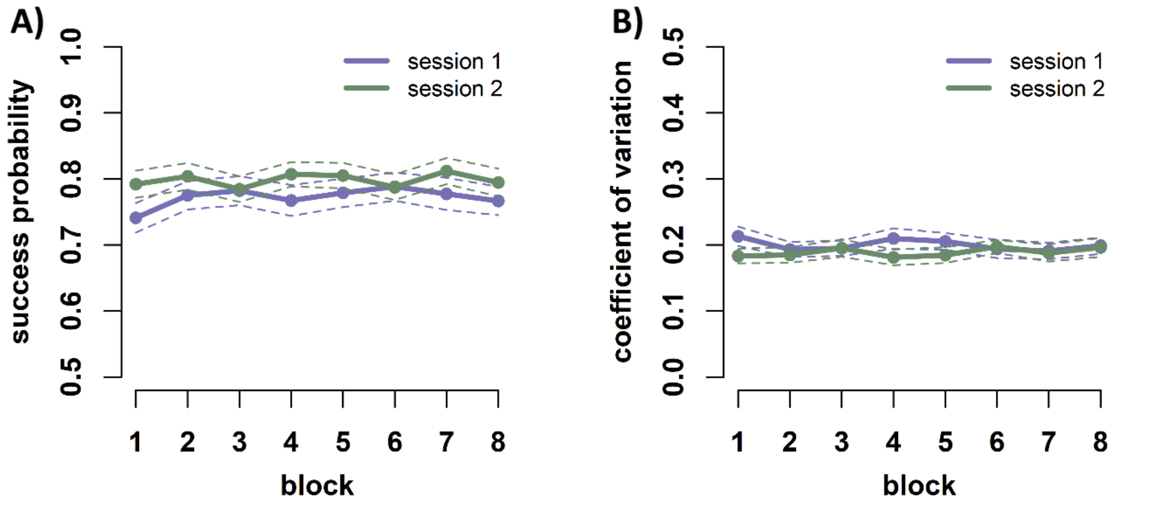

Figure S3. *Task performance remained consistently high throughout sessions.* **A)** *Success probability* of decisions slightly increased throughout sessions, with overall better decision performance in the second as compared to the first session. **B)** Variability of decisional performance, as indicated by the coefficient of variation of *success probability*, did not change throughout sessions, indicating that participants remained attentive throughout sessions. Variability was lower in the second as compared to the first session, indicating potentially better attention. Performance measures were computed per block (filled circles) and fitted with linear regressions per participant to statistically test changes over time (see text). Dashed lines reflect 95%-Confidence Intervals.

Blockwise session instructions were directed either towards bananas or caterpillars, as visually distinct representations of decision and movement phases. Participants were furthermore instructed with text and animations that the speed with which decisions and finger tapping movements were completed was inconsequential for the duration of a trial (except for decision duration in fast-decision blocks, which shortened trial duration). The task was thus designed to ensure that coregulation effects were not incentivized in terms of capture rate (see Discussion in the main text) and to ensure that participants were aware of this fact. However, in case participants were unmotivated and hence inattentive to task instructions, participants may have applied similar strategies to all aspects of the task when asked to either ‘choose bananas fast’ or to ‘move the caterpillar fast’, leading to observed coregulation effects. To address these concerns, in the subsequent paragraphs we demonstrate

that poor motivation or inattention to task instructions likely did not drive coregulation effects.

Behavior indicates that participants were motivated to perform well

Behavioral data is difficult to reconcile with the idea that participants were unmotivated: Almost all participants completed both sessions entirely (55 out of 62) and the ones that didn't faced mostly technical issues excluding them (see Participants in the main manuscript). Behavioral performance was high with an average of 85% correct decisions and 98% correct movements across all trials, although the majority of decisional stimuli were rather ambiguous (see Experimental task in the main manuscript) and movements could be constrained to specific speeds. Likewise, participants obtained block boni (which were given in decision sessions) in 90% of blocks on average, meaning that they likely aimed to follow task instructions. Moreover, although the Simple Reaction Time task was administered at the end of each session and entailed new instructions, 93% of SRT trials were performed correctly on average, suggesting that participants stayed motivated until the end of the session.

We also tested whether participant's performance changed throughout sessions, which may indicate a loss in motivation. For these tests, we focused on the *success probability* of decisions (i.e., the average probability that chosen bananas were the correct ones), since performance in the movement phase of the task was likely too high to show changes over time (see previous paragraph). As seen in Figure S3A, performance slightly increased throughout sessions. To test this statistically, we fit linear regressions to block performance for each participant and tested regression slopes with signed-rank tests against zero. Performance increased in session 1 (median increase per block = 0.4%, CI = 0.1 to 0.6%, S = 38, <u><</u>= .02) and in session 2 (median increase per block = 0.2%, CI = 0.0 to 0.4%, S = 38, p = .04). As seen in Figure S3B and probably more indicative of sustained attention (Cai et al.,

2021; Kelly et al., 2008; van Kempen et al., 2019), variability of performance did not significantly change in session 1 (median change per block = -0.1%, *CI* = -0.3 to 0.0%, S = 22, p = .09) and session 2 (median change per block = 0.1%, *CI* = 0.0 to 0.4%, S = 37, p = .07), as measured by the coefficient of variation of *success probability*. Importantly, signed-rank tests indicated that performance in session 2 was better as compared to session 1, since *success probability* was higher (median difference = 2.6 %, *CI* = 0.9 to 4.9%, S = 15, p = .01, see Figure S3A) and its variability was lower (median difference = 2.1%, *CI* = 1.0% to 3.4%, S = 38, p = .01, see Figure S3B). Taken together, this hence suggests that participants stayed motivated to perform well across sessions.

We also tested whether coregulation of decisions and movements depended on individual performance: If observed coregulation effects were primarily driven by unmotivated or inattentive participants, they should diminish in best performers. Therefore, for each session and based on *success probability*, we split participants into equally sized groups of best, intermediate and worst performers. As in the main manuscript, coregulation effects were defined as change in tapping duration from fast to slow decision-blocks, as well as change in decision duration from fast to slow tapping-blocks. For the decision session, we found highly significant coregulation effects across all three groups of performers (all p's $\leq 10^{-4}$), with no significant differences between groups as indicated by an ANOVA based on trimmed means ($F(2, 22.30) = 0.01$, p = .99). For the movement session, we found significant coregulation effects for best (p = .01) and intermediate performers (p = .02), but not for worst performers (p = .17), although the ANOVA indicated no significant differences between groups ($F(2, 21.89) = 0.76$, p = .48). These results hence provide strong evidence against the idea that coregulation effects were primarily driven by unmotivated and inattentive participants. Indeed, visual inspection of individual data in Figures 2B and 2E suggests that these effects were remarkably consistent on an interindividual level.

Participants who internalized instructions well showed clear coregulation effects

Table S2. *Instruction questionnaire.*

**decision session**
1. In 'accurate choice' segments, I felt like I could end a round earlier by choosing the banana faster.
2. In 'accurate choice' segments, I felt like I could end a round earlier by moving the caterpillar faster.
3. In 'fast choice' segments, I felt like I could end a round earlier by choosing the banana faster.
4. In 'fast choice' segments, I felt like I could end a round earlier by moving the caterpillar faster.
**movement session**
1. In segments in which the snake was retreating, I felt like I could end a round earlier by choosing the banana faster.
2. In segments in which the snake was retreating, I felt like I could end a round earlier by moving the caterpillar faster.
3. In segments in which the snake was chasing me, I felt like I could end a round earlier by choosing the banana faster.
4. In segments in which the snake was chasing me, I felt like I could end a round earlier by moving the caterpillar faster.

Note. Participants were asked to strongly agree, somewhat agree, somewhat disagree, strongly disagree or indicate that they 'don't know' to these statements after the experimental task in the respective session.

To probe whether participants internalized instructions, a short instruction questionnaire was administered after the experimental task in each session (see Table S2). In the decision session, one statement read: *"In 'fast-decision segments', I felt like I could end a round earlier by moving the caterpillar faster."* This statement was critical since faster finger tapping was observed in fast-decision blocks, although only fast decisions could shorten trial duration, allowing to perform more trials. Therefore, a proper understanding of the task design should lead participants to disagree with this statement. Surprisingly, as majority, 37 participants agreed with the statement, suggesting that deciding faster was associated with a subjective feeling of saving time through movement. Fifteen participants correctly disagreed with this statement, whereas six participants did not know. More importantly however, all participants, i.e. those who (somewhat or strongly) agreed (*median* = 18 ms, $S = 1$, $p = 10^{-8}$), (somewhat or strongly) disagreed (*median* = 14 ms, $S = 0$, $p = .001$) or did not know (*median*

= 10 ms, *S* = 0, *p* = .03), tapped faster in fast-decision blocks, with no significant difference between the three groups (*F*(2, 10.98) = 3.45, *p* = .07), as suggested by an ANOVA based on trimmed means with INSTRUCTION AGREEMENT as between-subjects factor and BLOCK TYPE as within-subjects factor. This suggests that deciding faster may have led to a subjective feeling of saving time by tapping faster in most participants. However, internalization of instructions was not a critical factor for the experimental effect observed in the decision session.

For the movement session, proper instruction comprehension was less of a concern as movement requirements were clearly constrained to the movement phase by visualizing them as retreating or chasing snakes (see Figure 1). Nevertheless, also here we probed internalization of the task design to test whether it modulated the experimental effect. The statement *"In segments in which the snake was chasing me, I felt like I could end a round earlier by choosing the banana faster."* was critical since participants performed faster decisions in these types of trials although this could not increase capture rate (see Discussion in the main text). A proper understanding of the task design should therefore lead participants to disagree with this statement. Twentynine participants correctly disagreed, 21 falsely agreed and nine did not know. More importantly however, faster decisions in fast-tapping blocks were observed in those participants who correctly disagreed (*median* = 94 ms, *S* = 4, *p* = .001), whereas there was no significant effect for those participants who agreed (*median* = 24 ms, *S* = 6, *p* = .16) or did not know (*median* = 95 ms, *S* = 2, *p* = .18). However, there was no significant INSTRUCTION AGREEMENT x BLOCK TYPE interaction (*F*(2, 19.62) = 0.78, *p* = .47), indicating that there was no significant difference in effect size between groups. This suggests that fast finger tapping may have led to a subjective feeling of saving time by deciding faster in a minority of participants. However, internalization of instructions was not a critical factor for the experimental effect observed in the movement session, as a clear

experimental effect was observed for the majority of participants who had a good comprehension of the task design.

Although less of an issue given this evidence, it may be a remaining concern that not all participants were able to restate instructions correctly. We believe that this was likely driven by the way that participants were asked to recall instructions: Participants had to (dis)agree with a set of statements which were probing whether participant's *subjective feelings regarding their own behavior* aligned with instructions. To probe this internalization of instructions, statements hence asked participants how they felt about the effectiveness of their own behavior (see Table S2). Agreeing with these statements is incompatible with the task instructions but may indicate that participants felt *as if* coregulating decisions and movements was somehow effective in speeding up a trial. Additionally, these statements were somewhat of "trick questions", since they were hidden among distractor statements and required participant's disagreement as correct answers. Indeed, the remarkable consistency of coregulation effects on an interindividual level as seen in Figure 2B and 2E despite divergent recall of instructions also speaks to the notion that a (mis)understanding of instructions was not driving behavioral findings.

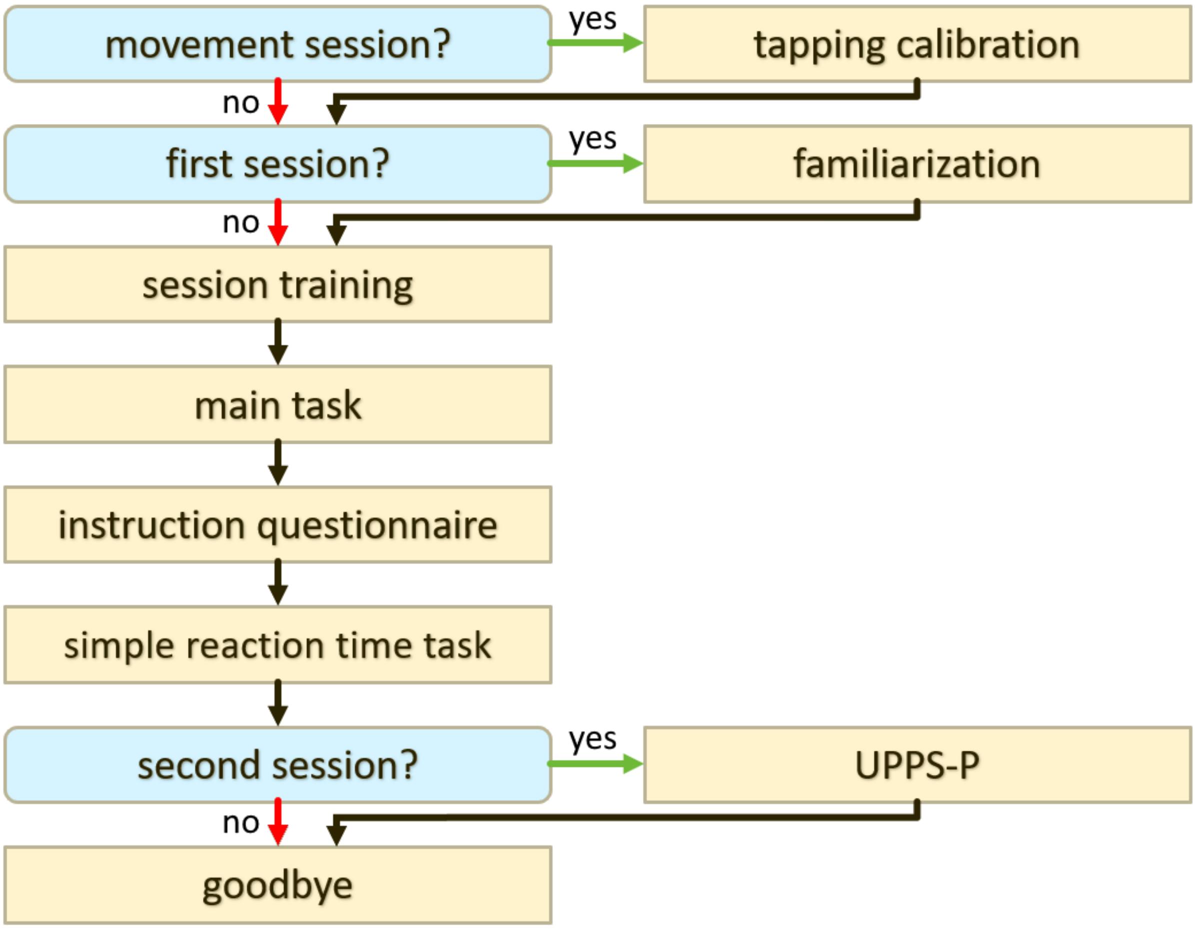

Figure S4. *Session outline.*

Behavior in the Simple Reaction Time task

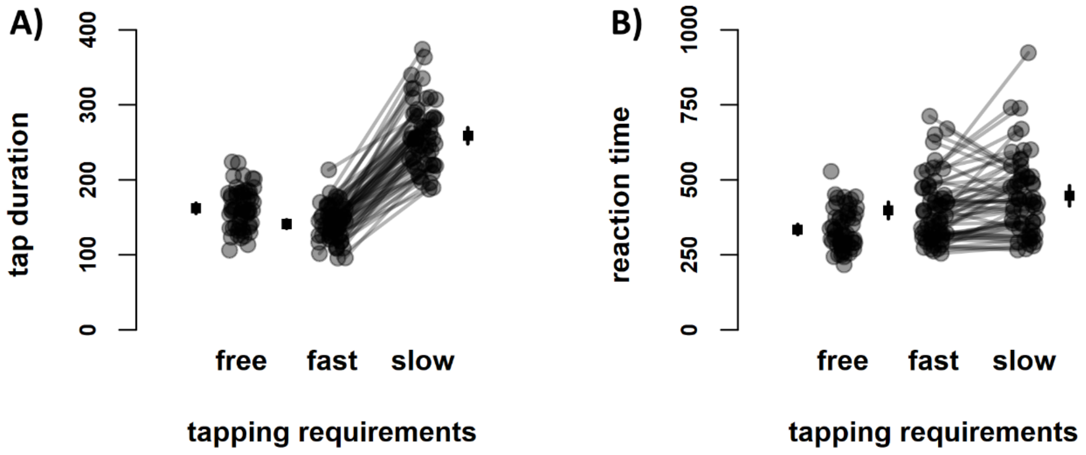

Figure S5. *Behavior in the simple reaction time task.* A simple reaction time task with no decisional aspects was administered, in which participants were required to tap with no speed constraints ('free', in decision session), or fast or slowly (in movement session). **A)** Tap duration was shortest when fast tapping was required, followed by free-tapping and slow-tapping blocks. **B)** Simple reaction times were fastest when no requirements on tapping speed were given, followed by fast-tapping and slow-tapping blocks. **A), B)** Individual data points are shown, with data stemming from the movement session linked with a line. Individual data points (circles) are flanked by group means (squares). Error bars of group means reflect 95%-Confidence Intervals.

Participants performed a simple reaction time (SRT) task, which was comparable to the main task, but stripped of decisional aspects. This allowed to estimate sensorimotor delays needed to perform a first key tap in order to isolate decision duration in the main task. The decision session entailed 40 SRT trials with no restrictions on tapping speed ('free' in Figure S5, no snake in the movement phase), whereas the movement session entailed two times 40 SRT trials requiring fast (chasing snake) or slow tapping (retreating snake). The effect of tapping requirements on tap duration and reaction times was tested in two separate repeated-measures ANOVAs for trimmed means (as more robust alternative to a conventional one-way repeated-measures ANOVA). Follow-up tests were conducted as signed-rank tests and corrected for multiple comparisons with the Bonferroni-Holm method (Chen et al., 2017). As intended, tap duration in the SRT task changed with tapping requirements ($F(1.61, 51.44) = 394.83$ and $p = 10^{-17}$). As seen in Figure S5A, tap duration

was shortest when fast tapping was required (*median* = 139 ms, *MAD* = 20 ms), followed by free tapping (*median* = 160 ms, *MAD* = 31 ms) and longest for slow tapping (*median* = 255 ms, *MAD* = 40 ms, all p's ≤ 10^{-9}). As seen in Figure S5B, reaction time also varied as a function of tapping requirements ($F(1.86, 59.59) = 30.32$ and $p = 10^{-8}$). Reaction time was shorter when fast tapping (*median* = 365 ms, *MAD* = 92 ms) was required as compared to slow tapping (*median* = 433 ms, *MAD* = 116 ms, $p = .001$). Interestingly, reaction time was shortest for free tapping (*median* = 315 ms, *MAD* = 71 ms. both p's ≤ 10^{-6}). Taken together, participants followed movement instructions well in the SRT task. Results further suggest that finger tapping with restrictions on the pacing (i.e. 'fast' or 'slow') takes more time to prepare than finger tapping with no speed requirements (i.e. 'free').

Since the SRT task was used to isolate decision durations in the main task by estimating sensorimotor delays under different tapping constraints, it is important to ensure that tapping behavior was comparable between SRT and main tasks for both sessions. For the decision session, we tested this by comparing average tap durations in the SRT task with those of the main task in a signed-rank test. Tap duration was 14 ms (*CI* = 10 to 18 ms) faster in the SRT task than in the main task ($S = 47$, $p = 10^{-7}$). For the movement session, we tested this in a repeated-measures ANOVA with the factors TASK and TAPPING REQUIREMENTS. In addition to the expected main effect of tapping requirements ($F(1,54) = 735.10$, $p = 10^{-32}$, $\eta^2_p = 0.93$), indicating faster tapping when fast tapping was required, there was a main effect of TASK ($F(1,54) = 21.95$, $p = 10^{-4}$, $\eta^2_p = 0.29$), indicating that participants tapped 6 ms (*CI* = 5 to 9 ms) faster in the SRT task than in the main task. Importantly however, there was no interaction between TASK and TAPPING REQUIREMENTS ($F(1,54) = 0.66$, $p = .42$, $\eta^2_p = 0.01$), suggesting that the change in tapping speed from fast-tapping to slow-tapping blocks was comparable between both tasks. In sum, participants tapped slightly faster in the SRT task than in the main task, potentially since the SRT task was administered at the end of each

session. Apart from that difference, tapping requirements induced highly comparable tapping behavior across tasks, suggesting that the SRT task was well-suited to estimate sensorimotor delays of the main task.

Effect of trial difficulty on behavior

To ensure that the difficulty of decisions remained comparable, each block contained 30 % obvious, 30 % ambiguous, 20 % misleading and 20 % random trials (see Experimental task in the main manuscript). This ensured that participants remained engaged and were not able to time their responses in advance. To test whether main findings reported in the main manuscript depended on trial difficulty, for each session we conducted a repeated-measures ANOVA. Taking either tap duration (decision session) or decision duration (movement session) as dependent variables, we tested the effects of factors EXPERIMENTAL CONDITION and TRIAL DIFFICULTY. Follow-up tests were conducted as Holm-corrected paired-samples *t*-tests. For the decision session, we found that tap duration relied on EXPERIMENTAL CONDITION ($F(1,56) = 57.30$, $p = 10^{-9}$, $\eta^2_{p} = 0.51$), as well as on TRIAL DIFFICULTY ($F(2.44,136.40) = 3.11$, $p = .04$, $\eta^2_{p} = 0.05$). However, there was no interaction between the two factors ($F(2,111.90) = 2.62$, $p = .08$, $\eta^2_{p} = .05$). Whereas the effect of EXPERIMENTAL CONDITION was expected given the findings reported in the main manuscript, the effect of TRIAL DIFFICULTY stemmed from the fact that participants tapped minimally faster in ambiguous trials (*median* = 181 ms, *MAD* = 26 ms) as compared to obvious ones (*median* = 182 ms, *MAD* = 29 ms, $p = .01$), with no other significant comparisons (all p's $\geq .09$). For the movement session, again as expected we found that decision duration relied on EXPERIMENTAL CONDITION ($F(1,58) = 9.84$, $p = .01$, $\eta^2_{p} = 0.14$). We also found that decision duration depended on trial difficulty ($F(1.85,107.10) = 421.20$, $p = 10^{-49}$, $\eta^2_{p} = 0.88$). Follow-up tests indicated that decision duration differed between each level of TRIAL DIFFICULTY (all p's $\leq 10^{-4}$): Decision duration was circa 1156 ms in obvious trials (*MAD* = 343 ms), circa 1617 ms in random trials (*MAD* = 326 ms), 1760 ms in misleading trials (*MAD* = 359 ms) and 1775 ms in ambiguous trials (*MAD* = 235 ms). There was also a significant interaction between EXPERIMENTAL CONDITION and TRIAL DIFFICULTY ($F(3,174) = 4.62$, $p = .01$, $\eta^2_{p} = 0.07$). Follow-up tests indicated that decision

duration significantly differed between fast-tapping and slow-tapping blocks in obvious (p = .01, *median* = 88 ms, *MAD* = 145 ms), random (p = .01, *median* = 102 ms, *MAD* = 132 ms) and misleading (p = .01, *median* = 59 ms, *MAD* = 141 ms) trials, but not in ambiguous trials (p = .18, *median* = 44 ms, *MAD* = 97 ms).

Taken together, trial difficulty affected behavior, however experimental effects were consistent across multiple levels of trial difficulty. Hence, these Supplemental Results do not alter conclusions drawn in the main manuscript.

How success probability of decisions is computed

As an objective measure of the quality of decisions, success probability was computed. In each trial of our task, evidence continued to change dynamically after the decision. Therefore, decisions had a probability of being correct, corresponding to the objective probability that the chosen banana would be longest by the end of the trial. Therefore, success probability of each decision can be formally calculated as the proportion of all hypothetical growth patterns of bananas following the decision in which the chosen banana wins. In accordance with previous work (Derosiere et al., 2019; Reynaud et al., 2020; Saleri Lunazzi et al., 2021; Thura & Cisek, 2014, 2016, 2017), success probability can therefore be computed as

$$p(S|N_O, N_N) = \frac{N_O!}{2^{N_O}} \sum_{f=0}^{\min(N_O, 89-N_N)} \frac{1}{f!\,(N_O - f)!}$$

where the probability *p* of the selected banana *S* winning is equivalent to the probability of unselected banana *N* not winning. N_N is the number of frames in favor of the unselected banana, N_O is the number of outstanding frames, both at the estimated time of decision offset (i.e. ‘decision duration’). The selected banana *S* wins if the number of frames in favor of the unselected banana *N* stay below 89, i.e. less than half of the 179 frames. The success probability of the selected banana therefore depends on the total number of possible growth patterns for the remaining frames N_O, in which the number of frames in favor of the unselected banana remain below 89, relative to the total number of all hypothetically possible growth patterns for these frames.

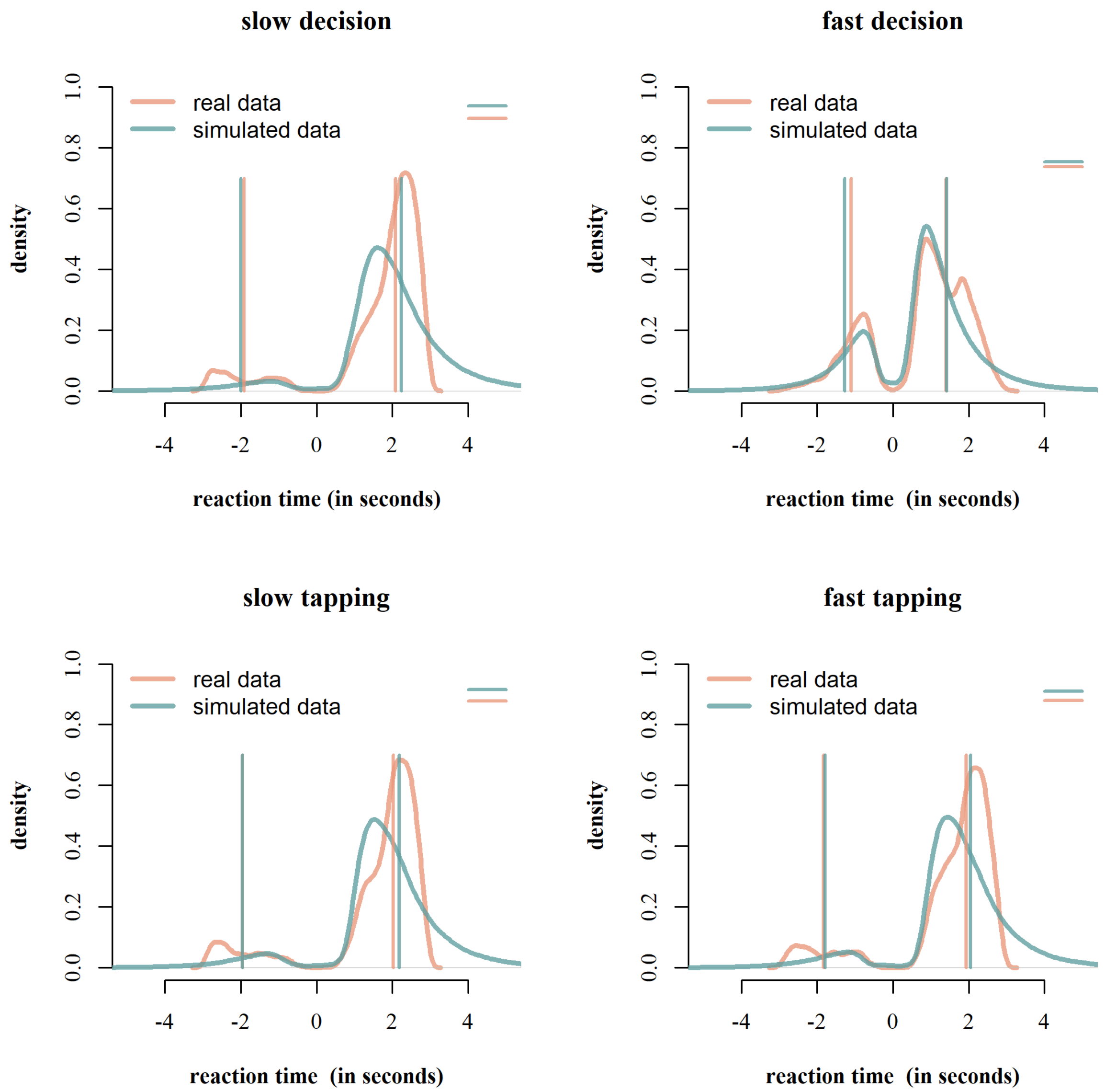

Figure S6. *Comparison between empirical data and data as predicted by the best-fitting drift diffusion models of each session.* Density distributions are plotted with a fixed bandwidth of 100 ms. Negative reaction times reflect incorrect decisions. Fitted models predict mean reaction times in the decision phase of the task (vertical bars) and proportion of correct decisions (horizontal bars in the upper right corner of each plot, scale from 0 and 1) of each experimental condition well, despite of a slight tendency to overestimate accuracy. Importantly, fitted models predict a reduction in reaction times for both correct and incorrect decisions in both fast-decision and fast-tapping blocks as compared to slow-decision and slow-tapping blocks, as observed in empirical data. As such, behavioral differences in reaction times observed in the decision phases of experimental conditions were replicated well, which was the effect of interest. Therefore, agreement between observed and simulated data was considered satisfactory for the purposes of this study. However, whereas early responses show a strong agreement between simulated and real data, late responses do not: In this experiment, by design it was advantageous to respond shortly before the timeout of three seconds to maximize success probability, causing shifted reaction time distributions towards late responses in real data. The drift diffusion model, 'blind' to this deadline, simulates a distribution with late responses exceeding three seconds, thereby underestimating the high

proportion of responses just before timeout. Remarkably, data in fast-decision blocks pose the exception, as here real and simulated data show a strong overlap. This is because only in fast-decision blocks choosing as soon as possible was the best strategy, leading to a low proportion of responses just before timeout. For each participant, the same number of trials were simulated as present in the real data. Data was simulated in this way 100 times, as if simulated participants performed the experiment 100 times. Resulting response distributions were averaged to produce most likely behavioral distributions for each model.

References

1. Chen, S.-Y., Feng, Z., & Yi, X. (2017). A general introduction to adjustment for multiple comparisons. *Journal of Thoracic Disease*, *9*(6), 1725–1729.
2. Derosiere, G., Thura, D., Cisek, P., & Duque, J. (2019). Motor cortex disruption delays motor processes but not deliberation about action choices. *Journal of Neurophysiology*, *122*(4), 1566–1577.
3. Reynaud, A. J., Saleri Lunazzi, C., & Thura, D. (2020). Humans sacrifice decision-making for action execution when a demanding control of movement is required. *Journal of Neurophysiology*, *124*(2), 497–509.
4. Saleri Lunazzi, C., Reynaud, A. J., & Thura, D. (2021). Dissociating the Impact of Movement Time and Energy Costs on Decision-Making and Action Initiation in Humans. *Front Hum Neurosci*, *15*. PubMed Central. http://europepmc.org/abstract/PMC/PMC8592235
5. Thura, D., & Cisek, P. (2014). Deliberation and commitment in the premotor and primary motor cortex during dynamic decision making. *Neuron*, *81*(6), 1401–1416.
6. Thura, D., & Cisek, P. (2016). Modulation of premotor and primary motor cortical activity during volitional adjustments of speed-accuracy trade-offs. *Journal of Neuroscience*, *36*(3), 938–956.
7. Thura, D., & Cisek, P. (2017). The basal ganglia do not select reach targets but control the urgency of commitment. *Neuron*, *95*(5), 1160–1170.
